# Simple and Effective HPLC Method for Elucidating Glycerol Oxidation Products

**DOI:** 10.1021/acsmeasuresciau.5c00024

**Published:** 2025-05-31

**Authors:** Eva Ng, Camilo A. Mesa, Elena Mas-Marzá, Sixto Giménez

**Affiliations:** † Institute of Advanced Materials, 16748 Universitat Jaume I, 12006 Castelló, Spain; ‡ Catalan Institute of Nanoscience and Nanotechnology (ICN2), CSIC, Barcelona Institute of Science and Technology, UAB Campus, 08193 Bellaterra, Barcelona, Spain

**Keywords:** Glycerol electrooxidation, high performance liquid chromatography, dihydroxyacetone, product quantification, validation

## Abstract

The glycerol electrooxidation reaction (GEOR) has been gaining increasing attention as a substitute for the oxygen evolution reaction to improve H_2_ production while producing high-value-added products. During GEOR, several C_3_, C_2_, and C_1_ species can be generated, making the detection and quantification of all these products a complex challenge that has not been fully addressed yet. Our study describes the development and optimization of a simple high-performance liquid chromatography (HPLC) method, capable not only of detecting but also simultaneously quantifying eight different GEOR products using a single diode array detector (DAD). To address possible overlapping signals, an indirect quantification approach is also proposed. The optimized method has been applied to real electrochemical GEOR systems, employing a Ni foil in alkaline media or a Pt foil in acidic media as oxidation electrocatalysts. Results show how product distributions varied significantly along with the pH, with formate being the main product in alkaline conditions (∼68% selectivity), whereas glyceraldehyde and dihydroxyacetone were the major products in acidic conditions (∼40% and ∼26%, respectively).

## Graphical abstract

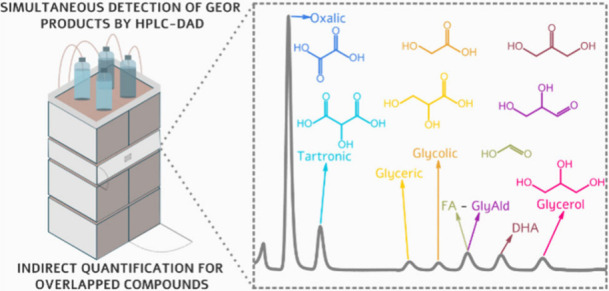

## Introduction

The glycerol electrooxidation reaction (GEOR) has gained significant attention as a sustainable alternative to the anodic oxygen evolution reaction (OER) in electrochemical systems.^1,2^ Coupling GEOR with the hydrogen evolution reaction not only facilitates H_2_ production as a fuel candidate but, more recently, the simultaneous integration of GEOR with the electrochemical reduction of CO_2_ has also been explored, enabling the cogeneration of valuable oxidation and reduction products.^3–6^ Moreover, glycerol electrooxidation results in the generation of high-value-added products such as glyceraldehyde (GlyAld), dihydroxyacetone (DHA), and formic acid.^7–9^ These products have applications in the chemical, pharmaceutical, and food industries, making GEOR a promising pathway for renewable energy and value-added chemical synthesis, compatible with circular economy schemes.^10,11^

Despite its potential, studying GEOR product distribution remains challenging due to the complexity of the reaction pathway, the diversity of possible products, and their similar chemical properties, which represent a significant difficulty to effective separation and subsequent accurate quantification.^12–14^ High-performance liquid chromatography (HPLC) has proven to be a powerful tool for separating, identifying, and quantifying small organic molecules, especially in aqueous media.^15–17^ However, conventional HPLC methods often face limitations in resolving all GEOR products simultaneously, commonly requiring refined equipment.^16,18,19^ Table 1 summarizes published HPLC parameters for the identification of glycerol oxidation products. While some of these works are not centered on analytical development, they provide a valuable context for evaluating existing strategies. A few recent studies involving GEOR-CO_2_RR coupling have also been included, as they contribute relevant insights into complex multiproduct electrochemical systems. For example, Goetz et al. reported the overlapping of peaks (i.e., DHA and glycerol), Kumari et al. and Kong et al. employed two different detectors, and Perini et al. employed several columns to fully separate the obtained products.^16,18–20^ More recently, Chiang’s group successfully employed HPLC-DAD for the detection and quantification of GEOR products across different catalytic systems.^21–23^ While these works demonstrate effective analytical strategies, their primary focus lies on the design and performance of the developed electrocatalysts. In most cases, the HPLC methods are not extensively discussed in terms of parameter optimization, validation, or resolution of overlapping compounds, whereas the temperatures employed in these methods are often significantly high (above 60 °C), which may compromise the stability of certain products during analysis (i.e., DHA, GlyAld, glyceric acid). Furthermore, these methods may lack versatility, making them unsuitable for a wide range of systems (e.g., pH-dependent measurements or various catalysts) in which selectivity can significantly change.

**Table 1:** Method Conditions Reported in the Literature Employing HPLC for the Analysis of GEOR, Highlighting the Primary Reaction Investigated in Each Study^a^

***Column***	***Injection volume (μL)***	***Flow rate*** (mL/min)	***Mobile phase***	***Oven temperature***	***RID temperature***	***DAD Wavelengths (nm)***	***Products***	***Study Focus***	***Reference***
**AS9 – HC**	50	0.6	H_2_SO_4_ 5 mM – ACN (94/6)	50	*--*	210	Mesoxalate, lactate, glyceraldehyde and glyceric acid	Coupled GEOR-CO_2_RR	^3^
**Hi-Plex-H, 300 × 7.7 mm**	50	0.6	H_2_SO_4_ 5 mM – ACN (94/6)	50	*--*	*--*	Formic, tartronic, glyceraldehyde and glyceric acid	Coupled GEOR-CO_2_RR	^4^
**ReproGel H (9.0 μm, 300 × 8 mm)**	*--*	0.5	H_2_SO_4_ 5 mM	55	*--*	*--*	Formate, tartronate, glycerate, formate, oxalate	Coupled GEOR-CO_2_RR	^5^
**Aminex HPX-87C**	20	0.5	H_2_SO_4_ 3 mM	70	30	210	GlyAld, DHA, glyceric acid, glycerol	GEOR	^25^
**Aminex HPX-87H**	--	0.5	H_2_SO_4_ 5 mM	65	--	--	Oxalic, formic, tartronic, glyceraldehyde, glycolic, glyceric acid	GEOR	^26^
**Hi-Plex H 7.7 × 300 mm**	20	--	H_2_SO_4_ 5 mM	65	--	--	Glyceric, glycolic and formic acid	GEOR	^27^
**Aminex HPX-87H**	20	0.5	H_2_SO_4_ 5 mM – ACN (65:35)	25	--	--	Glycerol, dihydroxyacetone (DHA), 3-hydroxy-propionaldehyde (3-HPA), and 1,3-propanediol (1,3-PD)	GEOR	^28^
**Zorbax SAX column**	--	1	H_3_PO_4_ (0.1% w/w) in H_2_O/acetonitrile (1/2 v/v)	25	--	210	Glycerol, glyceric and glycolic hydroxypyruvic acid; DHA and glyceraldehyde	GEOR	^29^
**Aminex HPX-87C**	10	0.5	H_2_SO_4_ 10 mM	60	--	--	Glycerol, glyceric acid and tartronic acid	GEOR	^30^
**Aminex HPX-87H**	10	--	H_2_SO_4_ 10 mM	--	--	--	Glycerol, DHA, glyceric and tartronic acid	GEOR	^31^
**Aminex HPX-87H**	40	0.4	H_2_SO_4_ 5 mM	60	--	--	Glycerol, glyceraldehyde, glyceric, tartronic and mesoxalic acid	GEOR	^32^
**Aminex HPX-87H**	--	0.6	H_2_SO_4_ 5 mM	55	--	--	Glyceric, glycolic, tartronic, oxalic, lactic	GEOR	^33^
**Aminex HPX-87H**	--	0.5	H_2_SO_4_ 5 mM	45	--	--		GEOR	^34^
**Aminex HPX-87H**	1	--	H_2_SO_4_ 5 mM	30 and 80	--	--	Glyceraldehyde, glyceric acid, glycolic acid, formic acid, oxalic acid, and tartronic acid	GEOR	^35^
**Alltech IOA 1000**	--	0.4	H_2_SO_4_ 5 mM	25	--	192	Glycerol, glyceraldehyde, DHA and formic acid (FA)	GEOR	^36^
**Alltech QA-1000**	20	--	H_2_SO_4_ 0.4 mM	70	--	--	Glycerol, glyceric, glyceraldehyde, tartronic, oxalic	GEOR	^37^
**Hitachi GL-C610-S**	5	0.45	H_2_O	60	--	210.8	Glycerol, DHA, glyceraldehyde, glyceric acid, hydroxypyruvic acid, tartronic acid, and oxalic acid	GEOR	^38^
**Aminex HPX-87H**	--	0.6	H_2_SO_4_ 4 mM	35	--	--	Tartronic, glyceric, glycolic, oxalic, formic	GEOR	^39^
**Aminex HPX-87H**	20	0.5	H_2_SO_4_ 5 mM	65	--	--	Oxalic, tartronic, glycolic, glyceraldehyde, glyceric	GEOR	^40–42^
**Eurokat H, Knauer**	--	0.6	H_2_SO_4_ 5 mM	70	--	220	Glyceric, formic, oxalic, acetic	GEOR	^19^
**Aminex HPX-87H**	--	--	H_2_SO_4_ 5 mM	70	70	--	Glyceraldehyde, DHA, formic, glyceric, glycolic, hydroxypyruvic acid	GEOR	^43^
**HyperREZ XP H+**		0.5	H_2_SO_4_ 5 mM	60			Formic acid, GlyAld, DHA and glycoaldehyde	GEOR	^20^
**One Aminex HPX-87H and three Shodex Sugar SH1011**	20	0.6	H_2_SO_4_ 0.5 mM	40	40	205 and 254	Glycerate, glycolate, lactate, formate	GEOR	^16^
**ICSep ICE-COREGEL87H3**	--	0.5	H_2_SO_4_ 0.1%	40	--	Multiple	Formate, tartronate, hydroxypyruvate, oxalate, glycolate, glycolaldehyde, glyceraldehyde, DHA	GEOR	^18^
**Ion exclusion (unknown brand)**	--	0.5	H_2_SO_4_ 5 mM	70	--	--	Formic, DHA, tartronic, glyceraldehyde, glycolic acid	GEOR	^21–23^

a For example, GEOR, CO_2_RR, or their coupling.

It is important to acknowledge that more advanced analytical approaches have been developed to address product distribution complexity in GEOR. For instance, Kirici et al. demonstrated the integration of proton nuclear magnetic spectroscopy (^1^H NMR) for detailed product detection and quantification.^24^ While these methods offer powerful capabilities, they often require specialized instrumentation. In contrast, the present method prioritizes simplicity and accessibility, serving as a practical and reliable tool for routine product analysis in GEOR.

Based on these previous efforts, our work aims to shorten the gap in the literature by presenting an analytical method specifically designed, optimized, and validated for GEOR systems. Unlike studies in which HPLC is used primarily to support catalyst evaluation, the focus here is on establishing a versatile and reproducible protocol that can accurately resolve and quantify a broad range of GEOR products under different reaction conditions. With the aim to address all these challenges, we have accurately optimized and validated an HPLC method capable of simultaneously detecting and quantifying eight different GEOR products (oxalic acid (OA), tartronic acid (TA), glyceric acid (GA), glycolic acid (GCA), formic acid (FA), GlyAld, and DHA, including glycerol itself, where high conversions can be monitored using a single diode array detector (DAD). This method overcomes signal overlap, specifically between glycerol and DHA as well as between FA and GlyAld. The latter approach resolves the overlapping peaks of DHA and glycerol, while the overlapped peaks of FA and GlyAld are quantified indirectly, ensuring an overall accurate analysis. Our optimized protocol was then applied to study real electrochemical systems under both alkaline and acidic conditions, using Ni foil and Pt foil as oxidation electrocatalysts, respectively. Pt and Ni catalysts have been widely studied in GEOR, where Ni exhibits a complex array of products that can be formed while Pt is known for its high selectivity toward high-value-added products (i.e., GlyAld and DHA).^11,44–47^ Our evaluation of product distributions in different pH environments reveal distinct selectivity trends, highlighting the influence of reaction conditions on GEOR pathways. We believe that this study provides a simple and effective approach, avoiding the need for more sophisticated and costly instrumentation for advancing GEOR research and contributes to understanding the complex interplay between catalyst, electrolyte, and product selectivity.

## Results and Discussion

### Selection of HPLC Conditions

Several mobile phases have been reported for the separation and analysis of glycerol oxidation derivatives, including H_2_SO_4_ at concentrations ranging from 0.4 to 10 mM, H_3_PO_4_, and water (Table 1). Similarly, flow rates have been reported in the range of 0.4 mL·min^–1^ to 1 mL·min^–1^. However, these conditions can be limited by the availability and specifications of the column. Columns commonly used for separating small organic compounds and alcohols are based on cation ligand exchange. Depending on their diameter and length, flow rate and temperature may vary to achieve optimal separation.

In this study, a proton ligand exchange Hi-Plex column (300 × 6.5 mm) was used with H_2_SO_4_ as the mobile phase. This column allows for the addition of modifiers such as acetonitrile (up to 30%) and alcohols (up to 5%). The column can stand temperatures ranging from 40 to 60 °C, and the maximum allowable flow rate is 0.7 mL·min^–1^. Considering these parameters, the range of possible conditions for efficient separation, particularly for resolving glycerol and DHA by using a single detector (DAD), is limited. Through systematic testing of different flow rates, mobile phase compositions, and column temperatures, we evaluated and optimized the effects of various HPLC parameters. These observations provide valuable insights into the development and optimization of analytical methods, which will benefit both current and future users.

The optimization process began by varying the concentration of H_2_SO_4_ in the mobile phase from 5 to 100 mM. As shown in Figure S1, increasing the acid concentration results in faster elution and improved peak resolution. However, when the concentration exceeds 30 mM, peaks elute closer, potentially compromising resolution, particularly at higher product concentrations. Conversely, at low acid concentrations (e.g., 5 mM), peak broadening occurs, which also negatively affects chromatographic resolution. Despite partial overlap between the peaks of GlyAld and GA, as well as DHA and glycerol (Figure S2), an acid concentration of 10 mM was chosen as optimal, as it provided sufficient separation to further deconvolute overlapping signals.

To address these challenges, the role of modifiers was investigated by adding acetonitrile (1–30% of the total flow rate) to the 10 mM H_2_SO_4_ mobile phase. By increasing the acetonitrile content, the retention times of GlyAld and DHA were shifted to longer times, improving separation. Figure S3 shows the acetonitrile effect in a solution containing GA, GlyAld, DHA, GCA, glycerol, and FA. At 5%, both GA and GlyAld exhibited overlapping features at ∼9.5 min, as did DHA and glycerol at ∼11.5 min. Upon the increasing of acetonitrile content, both the retention time and the relative retention times of the species begin to shift erratically, hindering a complete and successful separation. The effect of methanol as a modifier was also studied at concentrations ranging from 1% to 5%. However, methanol caused peak broadening and strong retention within the system, which hindered the re-equilibration of the column to baseline conditions before the next measurement (Figure S4). This behavior is likely due to the nature of the Hi-Plex column, which is employed for the separation of weak acids and alcohols; in this context, methanol as a mobile phase does not favorably influence the column’s retention characteristics.

The effect of flow rate was evaluated in the range of 0.2 mL·min^–1^ to 0.6 mL·min^–1^. It was observed that increasing the flow rate shortens run time, sharpens peaks, and reduces interpeak spacing (Figure S5). However, the latter might result in possible overlapping peaks. In general, it is important to maintain a compromise between interpeak spacing and resolution to ensure accurate separation within a reasonable analysis time. Furthermore, the effect of temperature was studied from 34 to 60 °C. Figure S6 revealed a trend similar to that shown with the flow rate effect. High temperatures lead to faster elutions but reduced interpeak spacing. Particularly, the optimal temperature chosen for our system was 34 °C to continue with the tests.

Additionally, different injection volumes (10 μL, 20 μL, and 40 μL) were tested. An injection volume of 10 μL resulted in poor signal intensity, thus making difficult the detection and quantification of compounds at low concentrations. Conversely, an injection volume of 40 μL produced excessively broad peaks, negatively affecting the chromatographic resolution. For this system, 20 μL was identified as the optimal injection volume (Figure S7). Based on these evaluations, the optimal conditions were determined to be a flow rate of 0.26 mL·min^–1^, a mobile phase composition of 70:30 H_2_SO_4_ (10 mM) and acetonitrile, respectively, a column temperature of 34 °C, and a wavelength of 205 and 300 nm (for GlyAld). These parameters provided effective separation of several glycerol electrooxidation products (glycerol, GlyAld, DHA, OA, TA, GA, GCA, and FA) within a 30 min run time (Figure S8).

### Validation Method

The developed method was validated following the *Validation of Analytical Procedures: Text and Methodology* (CPMP/ICH/381/95) guidelines,^48^ assessing the parameters of linearity, limit of detection (LOD), limit of quantification (LOQ), precision, and accuracy. Under the optimized HPLC conditions, GlyAld, DHA, glycerol, OA, TA, GA, and GCA were successfully separated (Figure S8). However, GlyAld and FA exhibited overlapping signals. This issue is addressed through an indirect calculation of formic acid using different detection wavelengths, as explained below. The retention times for OA, TA, GA, GCA, DHA, and glycerol were 12.37, 13.64, 17.13, 18.31, 20.76, and 22.46 min, respectively. FA and GlyAld both eluted at 19.46 min.

The relationship between the peak area (*A*) and concentration (*C*) for the different standards under the optimized method was evaluated. Calibration curves were obtained using the concentrations 0.1, 0.5, 1.0, 2.5, and 5.0 mM for each standard. Figure 1 shows the calibration curves with their respective linear regressions. Each data point includes error bars from three different measurements, representing the variability at each concentration level. Correlation coefficients (*R* ^2^) were greater than 0.998 for all measured compounds within the 0.1–5 mM range, indicating a strong linear relationship.^49^

**Figure 1:**
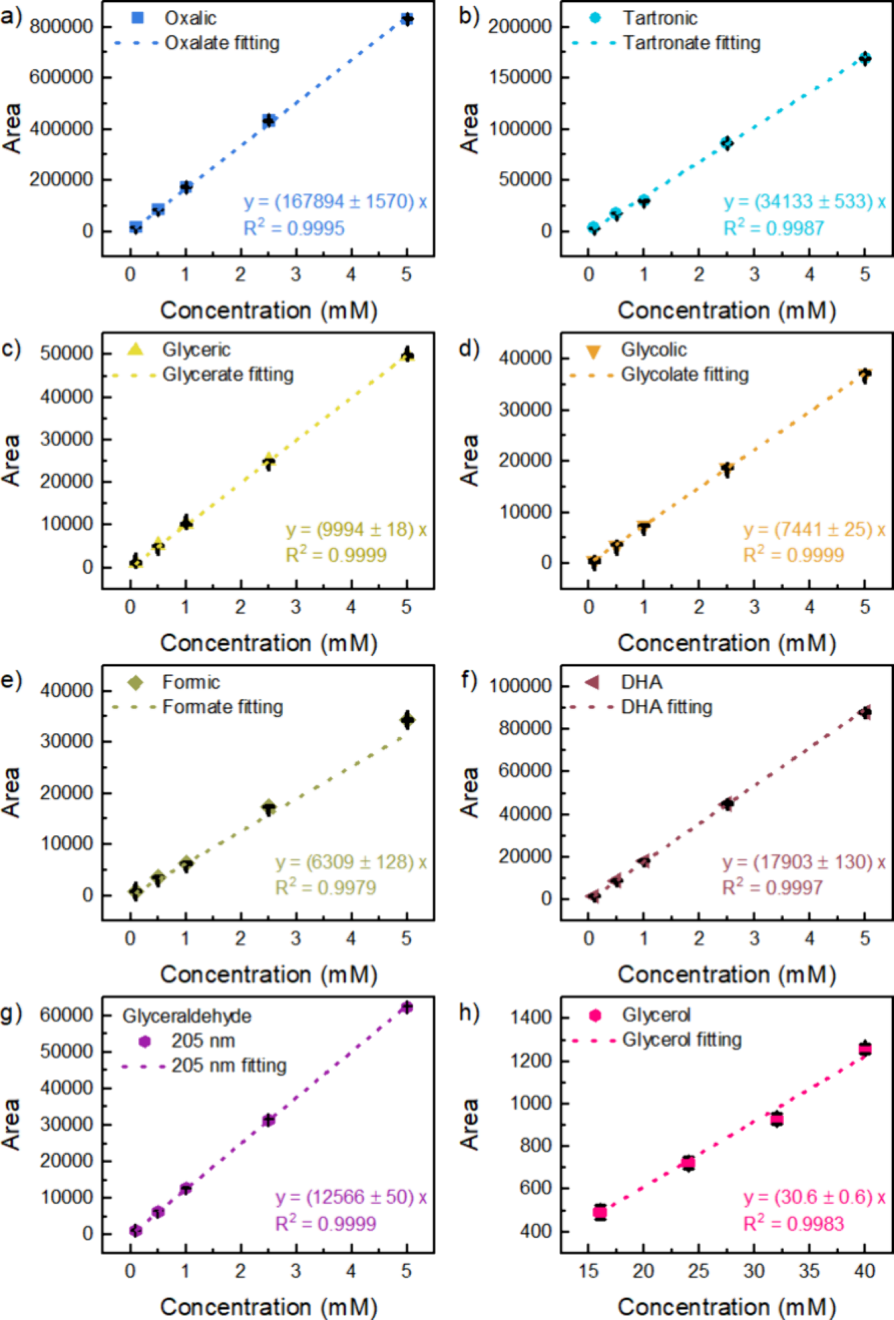
Calibration curves for a) OA, b) TA, c) GA, d) GCA, e) FA, f) DHA, g) GlyAld, and h) glycerol standards prepared in the mobile phase (10 mM H_2_SO_4_) from 0.1 to 5 mM and their linear regression, respectively. Each data point includes error bars representing the variability at each concentration level.

Furthermore, the LOD and LOQ were calculated using eq 2 and eq 3, respectively.

$$LOD=\frac{3.3\sigma }{S}$$ 1

$$LOQ=\frac{10\sigma }{S}$$ 2

where σ is the standard deviation of the response and *S* is the slope of the obtained curve. The LOD and LOQ values for the studied products are summarized in Table 2. Additionally, instrumental precision was assessed through repeatability, by measuring three different concentrations (0.5 2.5, and 5 mM) in triplicate for all glycerol products. The precision is expressed as the relative standard deviation (*RSD*), calculated using the standard deviation and mean concentration, as shown in eq 4.

$$RSD\left(\%\right)=\frac{\sigma }{\mathrm{C̅}}\times 100$$ 3

where σ represents the standard deviation of each measured concentration and *C̅* is the mean concentration. For all standards, except glycerol, the *RSD* was ≤1.61%. In the case of glycerol, the instrumental precision varied due to its poor absorbance. However, the precision for glycerol remained within acceptable limits, with an *RSD* ≤ 4.73%. Accuracy was evaluated by comparing the measured concentrations to the theoretical concentrations. These results are expressed as the recovery (*R*), determined by injecting the standard solutions three times at three concentrations (0.5 mM, 2.5 mM, and 5 mM), as calculated using eq 5.

$$R\left(\%\right)=\frac{C_{\exp}}{C_{theoretical}}\times 100$$ 4

The obtained accuracy was between (91 ± 4) % and (109.5 ± 0.5) %, and it is presented in detail for the studied products in Table 2 along with the precision data.

**Table 2:** Validation Method Parameters for Glycerol and the Different GEOR Products

**Parameters**	**Oxalic**	**Tartronic**	**Glyceric**	**Glycolic**	**Formic**	**DHA**	**GlyAld**	**Glycerol**
**Linearity range (*n* = 5) (mM)**	0.1–5	0.1–5	0.1–5	0.1–5	0.1–5	0.1–5	0.1–5	16–40
**Linear regression equation**	*y* = (167894 ± 1570)*x*	*y* = (34133 ± 533)*x*	*y* = (9994 ± 18)*x*	*y* = (7441 ± 25)*x*	*y* = (6309 ± 128)*x*	*y* = (17903 ± 130)*x*	*y* = (12566 ± 50)*x*	*y* = (30.6 ± 0.6)*x*
**Determination coefficient (R** ^**2**^ **)**	0.9996	0.9988	1.0000	0.9999	0.9979	0.9997	0.9999	0.9983
**LOD (mM)**	0.031	0.036	0.008	0.004	0.032	0.015	0.009	0.097
**LOQ (mM)**	0.094	0.109	0.024	0.012	0.096	0.044	0.028	0.293
**Repeatability (RSD, %)**	<5%							
*Lower (0.5 mM)*	0.48	0.35	1.07	0.96	1.61	0.35	0.49	4.73
*Middle (2.5 mM)*	0.56	0.16	0.17	0.21	0.46	0.23	0.23	3.35
*Highest (5 mM)*	0.11	0.34	0.33	0.23	0.33	0.12	0.14	1.37
**Recovery (R, %)**	∼70–120%							
*Lower*	102.3 ± 0.5	101.7 ± 0.4	96 ± 1	97.5 ± 0.9	111 ± 2	101.3 ± 0.4	103.8 ± 0.5	91 ± 4
*Middle*	102.6 ± 0.6	101.2 ± 0.2	98.8 ± 0.2	100.8 ± 0.2	109.5 ± 0.5	100.4 ± 0.2	100.1 ± 0.2	92 ± 3
*Highest*	98.6 ± 0.1	99.0 ± 0.3	98.7 ± 0.3	99.7 ± 0.2	108.4 ± 0.4	98.1 ± 0.1	98.3 ± 0.1	99 ± 1

### Indirect Determination of Formic Acid

As previously mentioned, during method optimization, the separation of two compounds out of eight was not achieved, despite the various conditions tested. GlyAld and FA exhibited overlapping signals at 19.46 min (Figure S9). However, GlyAld and FA concentrations were accurately quantified by using distinct detection wavelengths for GlyAld and implementing a simple, indirect method for FA quantification as explained below. Figure S10 represents three different concentrations (0.5, 2.5, and 5.0 mM) of glyceraldehyde standards measured at five different wavelengths (195, 205, 220, 270, and 300 nm). Each standard was analyzed three times to demonstrate reproducibility. Concentrations were calculated by using the corresponding slope at each wavelength, as shown in Table S1. For the method application, GlyAld was quantified directly at 300 nm, while the other compounds were measured at 205 nm, as detailed below.

Consequently, FA determination was performed by the linear regression equation given by eq 6.

y=mx+b 5

where *m* represents the slope, or observed extinction coefficient, which depends solely on the detector’s internal arrangement (e.g., light path length in the DAD), *x* the concentration, *b* the intercept and *y* the peak area (*A*). After determining the concentration of GlyAld at 300 nm, this value is used in eq 6 along with the observed extinction coefficient at 205 nm to calculate the expected peak area (*A*_*GlyAld*,205*nm*_). For a mixture of GlyAld and FA, their combined absorption at 205 nm can be expressed by eq 7. The peak area of formic acid (*A*_*FA*,205*nm*_) is then deduced, and, using its corresponding *m* at 205 nm, the unknown concentration of formic acid is calculated:

$$A_{GlyAld+FA}=A_{GlyAld,205nm}+A_{FA,205nm}$$ 6

To validate this approach, five mixtures of GlyAld and FA with equivalent concentrations (0.1, 0.5, 1.0, 2.5, and 5.0 mM) were prepared and measured three times, as shown in Figure 2. The *x*-axis represents the prepared standard solutions at different concentrations, while the *y*-axis represents the obtained concentration through the calibration curves. The gray pointed lines indicate a visual guide for the expected concentrations. The absolute error for each prepared solution was also calculated using eq 8:

$$Error\left(\%\right)=\left|\frac{E-T}{T}\right|\times 100$$ 7

where *E* represents the experimental value and *T* the theoretical value. All errors were below 10%, as summarized in Table 2. These results confirm that the indirect quantification of formic acid is a reliable method applicable not only to this specific system but also to others with similar characteristics.

**Figure 2:**
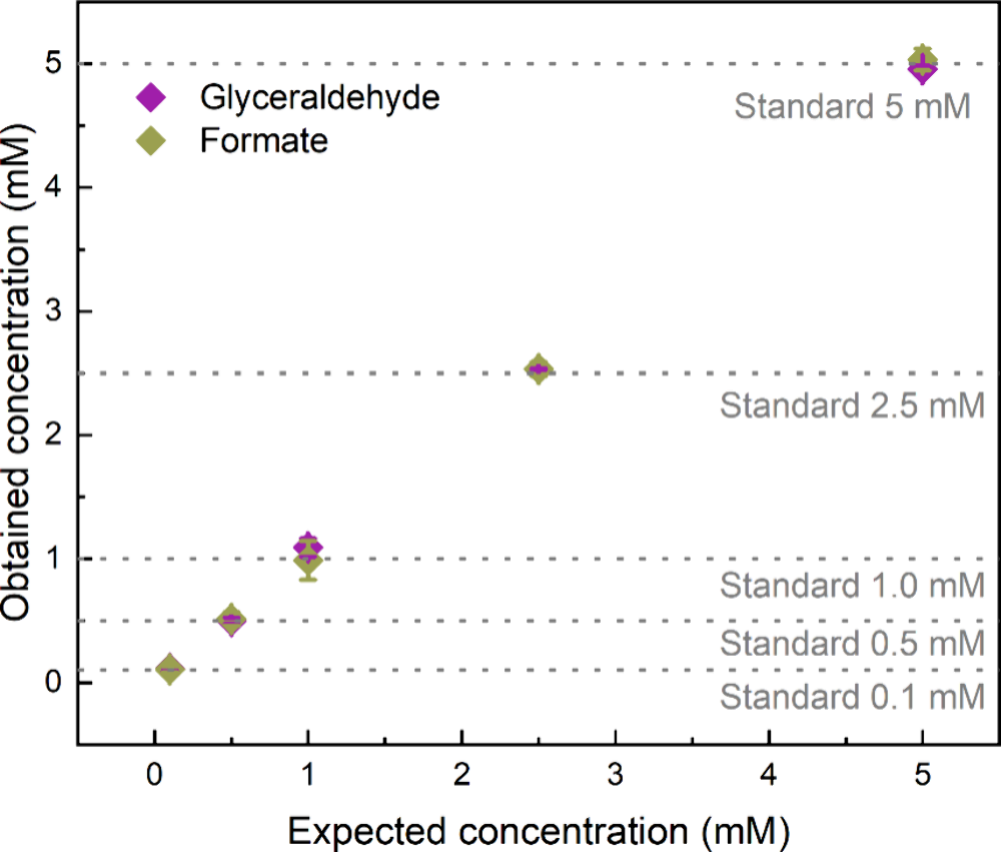
Obtained concentrations as a function of the expected concentration for glyceraldehyde and formic acid quantification with their standard deviation. Each measurement was performed three times.

### Application of the Method

To test this method in real electrochemical systems, potentiostatic measurements were conducted in both alkaline and acidic media using Ni foil and Pt foil as oxidation electrocatalysts, respectively. Suitable potentials for catalysis were identified using cyclic voltammetry (CV) (Figures S11 and S12). Under alkaline conditions, 0.1 M LiOH was used as the electrolyte due to its delayed onset potential for water oxidation compared to NaOH and KOH (Figure S13), with a glycerol concentration of 50 mM. The selected potential was within the region where the OER does not occur. Product distribution was analyzed at 1.62 V vs RHE until a charge of 50 C was passed (Figure S14). This charge corresponds to approximately 52% of the total required to transform 50 mM glycerol into DHA (a two-electron process), allowing for the identification of intermediate products prior to complete glycerol oxidation. Before HPLC analysis, all electrochemical samples collected in alkaline media were acidified to protect the column, which operates under protonated conditions. This acidification step neutralizes the matrix without affecting the analytes of interest. The only noticeable impact observed in the resulting chromatograms is the appearance of a salt-related peak at ∼11.38 min, which does not interfere with the resolution or quantification of GEOR products.

In acidic media, 0.5 M HClO_4_ was used as the supporting electrolyte to prevent sulfate adsorption onto the Pt foil electrode. A potential of 2 V vs RHE was selected for studying product distribution. Notably, from the CV shown in Figure S12, Pt exhibited higher activity toward the OER than the GEOR under these conditions. However, Pt was chosen for simplicity and to demonstrate the applicability of the developed HPLC method, given its ability to form DHA and GlyAld.^2,12,50^ Pulsed chronoamperometry was performed in a solution of 0.5 M HClO_4_ and 50 mM glycerol. Figure S15 compares regular and pulsed chronoamperometry, showing that the regular method exhibits current densities that are higher than those of the pulsed one. However, the Faradaic efficiency (FE) of the regular chronoamperometry was ∼11%, while that of the pulsed method was significantly higher, ∼60% (Figure S16). This indicates that a substantial portion of the system’s energy, in the regular method, is possibly consumed in the accumulation of poisoning intermediates or adsorption processes on the Pt surface.^5,51–57^ To mitigate this, a pulsed approach was used, applying 2 V vs RHE for 50 s, followed by a 10 s open circuit potential (OCP) period to desorb reversibly adsorbed products and minimize catalyst poisoning. The experiment was run for 2 h under these conditions (charge passed ∼ 40 C), as shown in Figure S15. A single sample was collected at the end of the experiment, and the corresponding chromatograms are presented in Figure 3.

**Figure 3:**
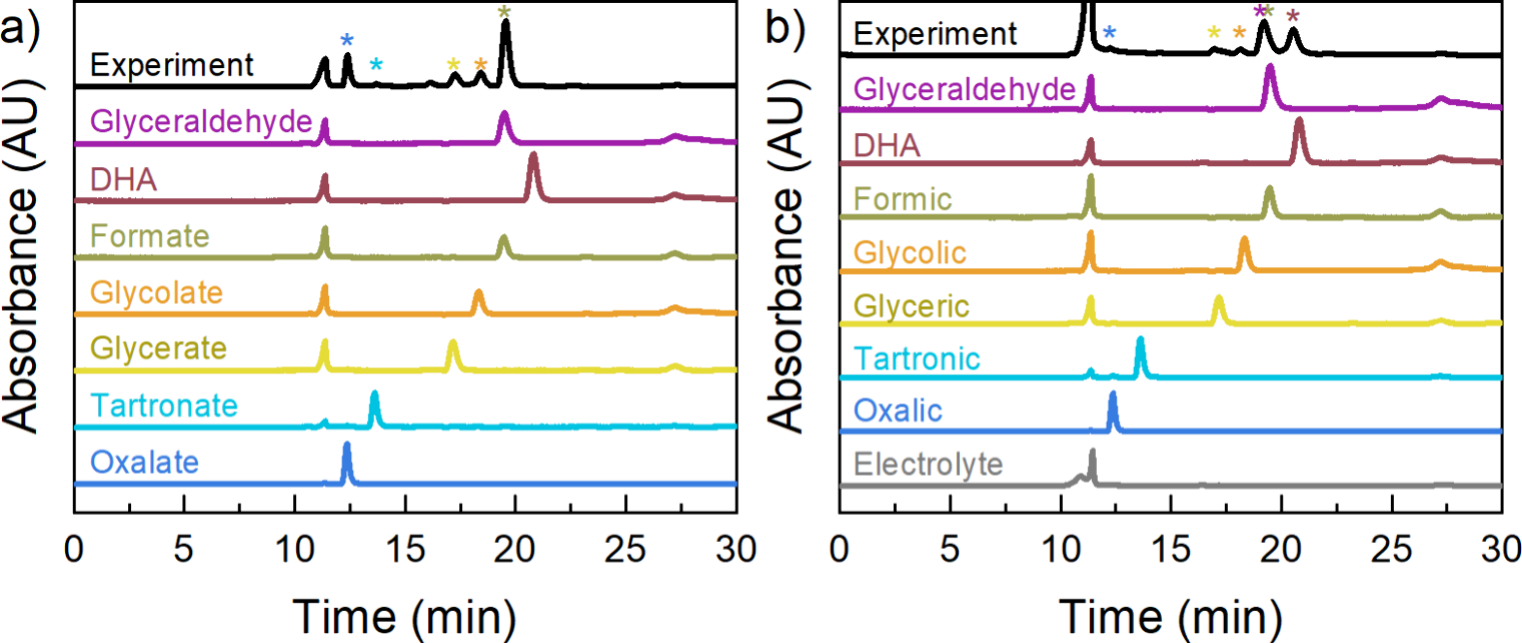
Chromatograms obtained from a potentiostatic measurement performed at a) 1.62 V vs RHE in a solution containing LiOH 0.1 M and glycerol 50 mM using Ni foil as electrode and at b) 2 V vs RHE in a solution containing HClO_4_ 0.5 M and glycerol 50 mM using Pt foil as electrode and applying pulsed chronoamperometry. Both figures contain the different standard chromatograms for reference. Colored symbols above the experimental curve represent the products obtained during the electrocatalysis.

Chromatograms from both alkaline and acidic media are compared to their respective standard chromatograms for reference. The results show that oxalate, tartronate, glycerate, glycolate, and formate were produced using the Ni foil catalyst in alkaline media (Figure 3a), while GlyAld, DHA, FA, GCA, and GA were produced in acidic media (Figure 3b). It is worth noting that the nomenclature of the products differs depending on the pH of the medium in which they were generated (i.e., “formate” in alkaline media versus “formic acid” in acidic media); however, changes in their retention times are negligible.

Both experimental chromatograms were evaluated at 205 and 300 nm to evaluate glyceraldehyde production. Figures S17 and S18 show the collected chromatograms at different wavelengths for the alkaline and acidic systems, respectively. In the alkaline system, as expected, no traces of GlyAld or DHA were observed due to their instability under highly oxidative conditions, which favor C–C bond cleavage.^7,18,58–60^ In contrast, in the acidic system, GlyAld and DHA were detected at 300 nm as products of the catalysis, consistent with findings reported in the literature.^24,61,62^

Product distribution in alkaline and acidic media was analyzed through selectivity, calculated using eq S9. Figure 4 illustrates the selectivity for the two electrodes. Pt-foil exhibited selectivities of approximately 40% and 26% toward GlyAld and DHA, respectively, with minor selectivities for GA (∼11%), GCA (∼8%), and FA (∼16%). Conversely, Ni-foil showed high selectivity toward formate (∼68%), followed by glycolate (∼16%) and glycerate (∼14%). Trace amounts of oxalate and tartronate were also detected, but their selectivity was below 2%. These experiments were performed three times to calculate each product variability.

**Figure 4:**
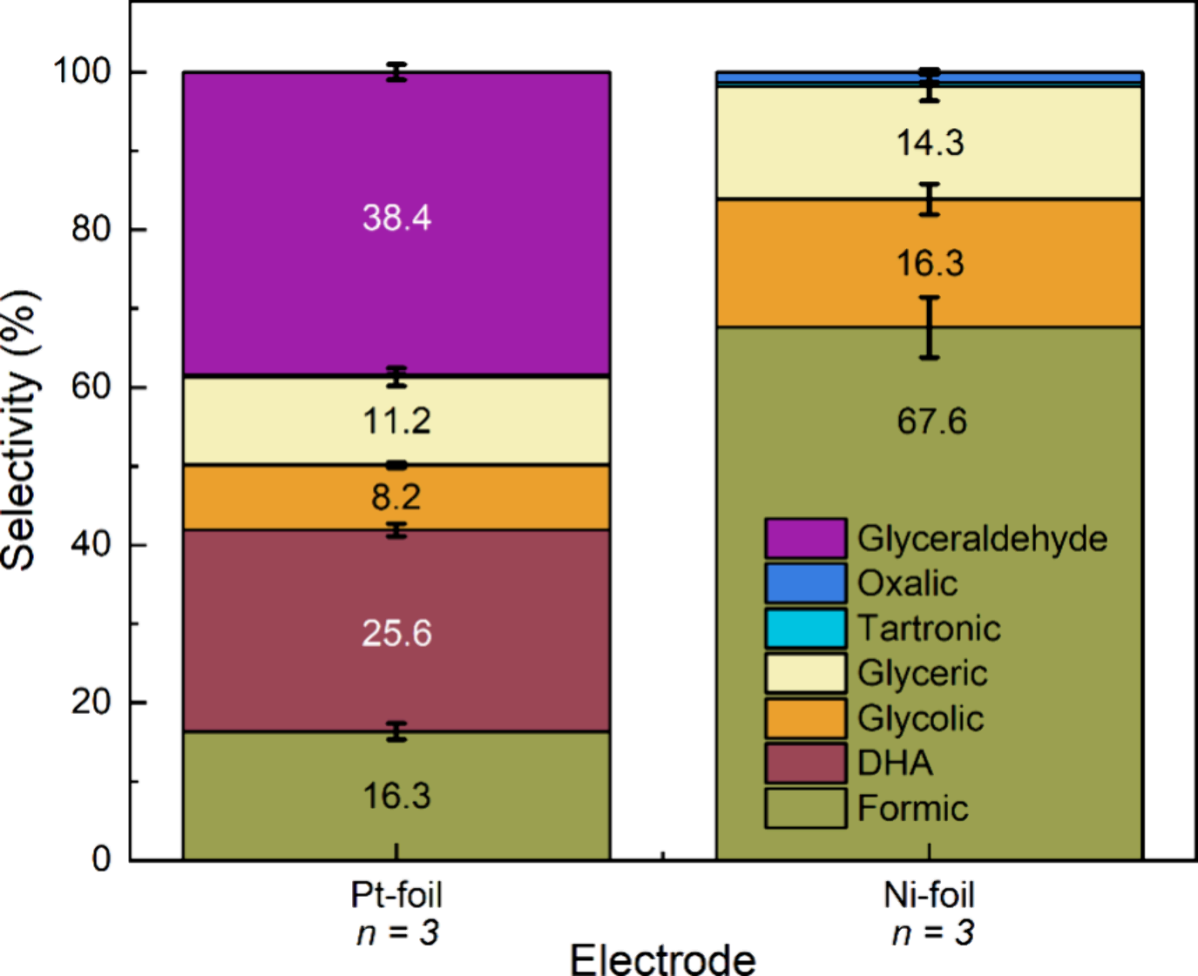
Product distribution is represented in terms of selectivity. The percentages inside the bars refer to each different product obtained. *n* refers to the repetitions of the experiment.

FE values for both Ni and Pt systems are presented in Figure S19. More detailed information about the FE calculation is provided in the Supporting Information. The alkaline system demonstrated an overall FE of ∼100%, while the acidic system showed an FE of ∼60%. Complete oxidation of glycerol may occur at high potentials (e.g., 2 V vs RHE with Pt foil), potentially leading to the evolution of CO_2_ and O_2_. These gaseous products are not detectable via HPLC, affecting the showed FE values.^18^ Moreover, the primary aim of this work is to develop a suitable HPLC method applicable to systems with varying product distributions under different experimental conditions, offering a straightforward approach to quantifying a wide range of glycerol electrooxidation products using a single detector. Additionally, as glycerol is completely separated from the rest of the products, its conversion and consumption during electrolysis can be easily addressed.

Recent studies performed under more industrially relevant conditions, such as high current densities, elevated temperatures, and extended electrolysis times, have reported product distributions that closely resemble those quantified in this study. For example, Balog et al. and Kormányos et al. observed formic, glycolic, glyceric, and tartronic acids as dominant oxidation products, even under strong oxidative conditions (≥200 mA·cm^–2^, up to 10 h of operation).^5,60^ These findings are aligned with our method, which addresses the reliable quantification of these key products. Moreover, minor products such as glyoxal, pyruvaldehyde, or lactate may also form under such conditions; however, our optimized protocol offers a solid foundation that could be adapted in future studies to incorporate such species, if needed.

For instance, lactic acid is a major concern since it is often produced across different photo- and electrochemical systems involving glycerol oxidation. For this reason, its detection was evaluated using individual calibration standards, and although it elutes close to glycolic acid (at 17.88 and 18.33 min, respectively), the two peaks are not fully resolved under the current conditions. This partial overlap may hinder direct quantification when both species coexist, since their separation through different wavelengths is not possible. However, the relative retention between them remains consistent across different concentrations (see Figure S20), enabling the application of signal deconvolution methods (e.g., modified Gaussian peak fitting) to distinguish and quantify these species when required.^63,64^

While this method was specifically developed for the analysis of GEOR products, the analytical approach, based on systematic HPLC optimization parameters, resolution of overlapping signals, and single detector quantification, may be extended to the study of other alcohols and polyols, which face similar analytical challenges in electrochemical oxidation processes, such as ethylene glycol, sorbitol, or mannitol electrooxidation.^65–67^

## Conclusions

This work presents a comprehensive study of an optimized HPLC method tailored to GEOR systems. The method allows for the simultaneous detection and quantification of a wide range of products, including glycerol, glyceraldehyde, DHA, formic acid, glycolic acid, glyceric acid, oxalic acid, and tartronic acid, produced under both alkaline and acidic conditions. By systematically investigating key parameters, such as mobile phase composition, flow rate, temperature, and injection volume, the method achieves high resolution, reproducibility, and reliability, ensuring accurate product identification and quantification. Furthermore, the method incorporates an easy approach to indirectly quantify formic acid, addressing challenges in overlapping signals using a single diode array detector.

The application of this method to real electrochemical systems demonstrates its robustness and versatility, highlighting its suitability for use in high-conversion glycerol systems. In alkaline media with a Ni-foil catalyst, the predominant products were formate, glycolate, and glycerate, showing a high selectivity toward C_1_ (∼67%) and C_2_ products (∼30%). Conversely, in acidic media with a Pt-foil catalyst, glyceraldehyde (∼38%) and DHA (∼25%) were the major products, showing the influence of pH and catalyst type on product distribution. Finally, this improved method provides a simple and effective approach for advancing GEOR research, serving as a guide to further developments and contributions to better understand the complexity of this reaction. Moreover, beyond its relevance to GEOR, the simplicity and versatility of the proposed HPLC method position it as a promising analytical tool for investigating the electrooxidation of other alcohols and polyols, given the common complexity of their product distributions and the similar analytical challenges that they present.

## Experimental Section

### Materials

All chemicals used are commercially available and were used without further purification: glycerol (≥99.5%, Sigma-Aldrich), LiOH monohydrate (99.995%, Sigma-Aldrich), perchloric acid (70%, Sigma-Aldrich), sulfuric acid (92%, 99.999% trace basis, Fisher Scientific), (l-glyceric acid sodium salt (≥95.0%, Sigma-Aldrich), glycolic acid (99%, Sigma-Aldrich), tartronic acid (≥97.0%, Sigma-Aldrich), formic acid (98–100%, Sigma-Aldrich), oxalic acid (98%, Sigma-Aldrich), d-(+)-glyceraldehyde (98%, Sigma-Aldrich), and dihydroxyacetone (Sigma-Aldrich). Ultrapure water (Milli-Q, resistivity >18 MΩ cm) was used to prepare all solutions.

As a working electrode (WE), nickel foil (99.0%, annealed, 0.2 mm thickness, GoodFellow) and platinum (Pt) foil (99.9%, 0.25 mm) were used. As the counter electrode (CE) and reference electrode (RE), a Pt wire and Ag/AgCl 3 M in KCl (Metrohm) were used, respectively.

For WE preparation, the nickel foil was cut into 3 cm × 1 cm strips and washed with aqua regia (HCl:HNO_3_ in 3:1 ratio) to dissolve any impurities, including organic and inorganic traces, as well as oxides, ensuring a uniform surface before each experiment and a systematic protocol. Then, the electrode was rinsed with Milli-Q water and masked with masking tape (3 M Company) exposing a one-side area of 2 cm^2^. Moreover, the Pt foil was cleaned with concentrated HNO_3_ to remove any impurity, including organic and inorganic traces. Then, the Pt foil was thoroughly rinsed with water. The area exposed for the reaction to occur was 5 cm^2^. All of the electrochemical measurements were normalized by the geometrical area since the electrodes are foils.

For the electrochemical cell, a single compartment cell made of borosilicate glass 3.3 was used to perform all of the electrochemical experiments. According to ISO 3585 and ISO 695-A2, the cell is suitable under the experimental conditions tested.^68,69^ Before each experiment, the electrochemical vessel was thoroughly washed with a solution containing KOH at 10 wt % in ethanol, rinsed with Milli-Q water, and then neutralized with HCl at 10% and rinsed again with Milli-Q water. This procedure is carried out to avoid any possible source of contamination.

### Electrochemical Measurements

The reaction performed by Ni-foil and Pt-foil as the catalysts were carried out in a VSP multichannel (BioLogic) and in an Autolab PGSTAT204 (Metrohm) potentiostats, respectively, using a three-electrode setup. Before the experiment, the Ni foil was activated in 0.1 M LiOH by performing 50 consecutive cyclic voltammetry scans at 100 mV/s under stirring. For the Pt-foil, 50 consecutive cyclic voltammetry scans at 200 mV/s and chronoamperometry at −0.5 V vs RHE for 60 s were performed in HClO_4_ 0.5 M as a cleaning procedure before each experiment. All electrolysis experiments were conducted under stirring to prevent mass transport limitations caused by glycerol diffusion. A 50 mM glycerol solution was prepared in either LiOH 0.1 M or HClO_4_ 0.5 M.

### Sample Treatment

For all electrolysis, aliquots of 1000 μL were taken, and 250 μL of H_2_SO_4_ 0.5 M was added in the case of the alkaline system to stop the reaction and ensure the stability of the formed products and for the acidic system to maintain the same procedure as before. Additionally, all the samples prior to being injected in the HPLC were filtered using a hydrophilic PTFE filter 13 mm, 0.22 μm.

### High Performance Liquid Chromatography (HPLC)

The electrolysis products were analyzed by means of high-performance liquid chromatography (HPLC, 1260 Infinity II, Agilent). The mobile phase was a 70:30 mixture of 10 mM H_2_SO_4_ (98%, HPLC grade) prepared with Milli-Q water and acetonitrile (HPLC grade). The stationary phase was a proton ligand exchange Hi-Plex column. The flow rate was 0.26 mL/min with a column temperature of 34 °C, and the injection volume was 20 μL. The equipment has a diode arrange detector (DAD) and a Refractive Index Detector (RID) as well. The wavelengths utilized in this work were 205 and 300 nm, where the latteris for glyceraldehyde quantification. Calibration curves of oxalic acid, tartronic acid, glyceric acid, glycolic acid, formic acid, glyceraldehyde, DHA, and glycerol were prepared and analyzed by triplicate. The glycerol consumption was not measured.

## Supplementary Material



## Notes

Faradaic efficiency (FE) equation and half-reaction involving transferred electrons for each compound; chromatograms for the systematic variation of HPLC parameters, slopes for GlyAld at different wavelengths, chromatograms regarding overlapped GlyAld and FA peaks, electrochemical measurements for Ni and Pt characterization and catalysis, FE of Ni and Pt catalysts, and lactic acid and GCA calibration curves (PDF)

## References

[1] De
Luna P., Hahn C., Higgins D., Jaffer S. A., Jaramillo T. F., Sargent E. H. (2019). What Would It Take for Renewably Powered Electrosynthesis
to Displace Petrochemical Processes?. Science.

[2] Luo H., Barrio J., Sunny N., Li A., Steier L., Shah N., Stephens I. E. L., Titirici M. M. (2021). Progress
and Perspectives
in Photo- and Electrochemical-Oxidation of Biomass for Sustainable
Chemicals and Hydrogen Production. Advanced
Energy Materials.

[3] Fernández-Caso K., Molera M., Andreu T., Solla-Gullón J., Montiel V., Díaz-Sainz G., Álvarez-Guerra M., Irabien A. (2024). Coupling Glycerol Oxidation
Reaction Using Ni-Co Foam
Anodes to CO2 Electroreduction in Gas-Phase for Continuous Co-Valorization. Chem. Eng. J..

[4] Fernández-Caso K., Peña-Rodríguez A., Solla-Gullón J., Montiel V., Díaz-Sainz G., Alvarez-Guerra M., Irabien A. (2023). Continuous Carbon Dioxide Electroreduction
to Formate
Coupled with the Single-Pass Glycerol Oxidation to High Value-Added
Products. J. CO2 Util..

[5] Kormányos A., Szirmai A., Endrődi B., Janáky C. (2024). Stable Operation
of Paired CO 2 Reduction/Glycerol Oxidation at High Current Density. ACS Catal..

[6] Wang G., Chen J., Li K., Huang J., Huang Y., Liu Y., Hu X., Zhao B., Yi L., Jones T. W., Wen Z. (2022). Cost-Effective
and Durable Electrocatalysts for Co-Electrolysis of
CO2 Conversion and Glycerol Upgrading. Nano
Energy.

[7] Houache M. S. E., Cossar E., Ntais S., Baranova E. A. (2018). Electrochemical
Modification of Nickel Surfaces for Efficient Glycerol Electrooxidation. J. Power Sources.

[8] Katryniok B., Kimura H., Skrzyńska E., Girardon J. S., Fongarland P., Capron M., Ducoulombier R., Mimura N., Paul S., Dumeignil F. (2011). Selective
Catalytic Oxidation of Glycerol: Perspectives
for High Value Chemicals. Green Chem..

[9] Zhang J., Shen Y. (2021). Electro-Oxidation of
Glycerol into Formic Acid by Nickel-Copper Electrocatalysts. J. Electrochem. Soc..

[10] Koranian P., Huang Q., Dalai A. K., Sammynaiken R. (2022). Chemicals
Production from Glycerol through Heterogeneous Catalysis: A Review. Catalysts.

[11] Hu X., Lu J., Liu Y., Chen L., Zhang X., Wang H. (2023). Sustainable
Catalytic Oxidation of Glycerol: A Review. Environ.
Chem. Lett..

[12] Tabassum N., Pothu R., Pattnaik A., Boddula R., Balla P., Gundeboyina R., Challa P., Rajesh R., Perugopu V., Mameda N., Radwan A. B., Abdullah A. M., Al-Qahtani N. (2022). Heterogeneous
Catalysts for Conversion of Biodiesel-Waste Glycerol into High-Added-Value
Chemicals. Catalysts..

[13] Sandid A., Spallina V., Esteban J. (2024). Glycerol to Value-Added Chemicals:
State of the Art and Advances in Reaction Engineering and Kinetic
Modelling. Fuel Process. Technol..

[14] Yin H., Yin H., Wang A., Shen L. (2018). Catalytic Conversion of Glycerol
to Lactic Acid over Graphite-Supported Nickel Nanoparticles and Reaction
Kinetics. J. Ind. Eng. Chem..

[15] Giertyas C. J., da Silva D. S., da Silva C. L. F., Meneghetti M. R., Plentz Meneghetti S. M., de Almeida R. M., Bortoluzzi J. H. (2019). Improvement
of an Analytical Method Based on HPLC with Refractive Index Detection
for the Analysis of Glycerol Oxidation Products. Quim. Nova.

[16] Perini N., Hessel C., Bott-Neto J. L., Pires C. T. G. V. M. T., Fernandez P. S., Sitta E. (2021). Photoelectrochemical
Oxidation of
Glycerol on Hematite: Thermal Effects, in Situ FTIR and Long-Term
HPLC Product Analysis. J. Solid State Electrochem..

[17] Ahmed M. A., Khan I., Hashim J., Musharraf S. G. (2015). Sensitive
Determination of Glycerol by Derivatization Using a HPLC-DAD Method
in Biodiesel Samples. Anal. Methods.

[18] Goetz M. K.
K., Bender M. T., Choi K.-S. (2022). Predictive Control of Selective Secondary
Alcohol Oxidation of Glycerol on NiOOH. Nat.
Commun..

[19] Kumari B., Braun M., Cychy S., Sanjuán I., Behrendt G., Behrens M., Muhler M., Andronescu C. (2023). Electrooxidation
of the Glycerol Derivative Solketal over Cu–Co Hydroxycarbonates
to Enable the Synthesis of Glyceric Acid. ChemElectroChem..

[20] Kong H., Gupta S., Pérez-Torres A.
F., Höhn C., Bogdanoff P., Mayer M. T., van de Krol R., Favaro M., Abdi F. F. (2024). Electrolyte Selection toward Efficient
Photoelectrochemical Glycerol Oxidation on BiVO4. Chem. Sci..

[21] Vo T. G., Tsai P. Y., Chiang C. Y. (2023). Tuning Selectivity and Activity of
the Electrochemical Glycerol Oxidation Reaction by Manipulating Morphology
and Exposed Facet of Spinel Cobalt Oxides. J.
Catal..

[22] Haryanto M. C., Hartanto R., Vo T. G., Chiang C. Y. (2024). Factors Affecting
Selective Electrochemical Glycerol Oxidation to Three-Carbon Products
over Cuprous Oxide Microcubes. J. Taiwan Inst.
Chem. Eng..

[23] Vo T.-G., Tran G.-S., Chiang C.-L., Lin Y.-G., Chang H.-E., Kuo H.-H., Chiang C.-Y., Hsu Y.-J. (2023). Au@NiSx Yolk@Shell
Nanostructures as Dual-Functional Electrocatalysts for Concomitant
Production of Value-Added Tartronic Acid and Hydrogen Fuel. Adv. Funct. Mater..

[24] Yelekli
Kirici E., Angizi S., Higgins D. (2024). A Universal Roadmap
for Quantification of Glycerol Electrooxidation Products Using Proton
Nuclear Magnetic Spectroscopy (1 H NMR). ACS
Catal..

[25] Beltrán-Prieto J. C., Pecha J., Kašpárková V., Kolomazník K. (2013). Development of an Hplc Method for the Determination
of Glycerol Oxidation Products. J. Liq. Chromatogr.
Relat. Technol..

[26] Zhang J., Shen Y., Li H. (2023). Electrolysis
of Glycerol by Non-Noble
Metal Hydroxides and Oxides. ACS Appl. Energy
Mater..

[27] Suzuki N. Y., Santiago P. V. B., Galhardo T. S., Carvalho W. A., Souza-Garcia J., Angelucci C. A. (2016). Insights of Glycerol Electrooxidation on Polycrystalline
Silver Electrode. J. Electroanal. Chem..

[28] Chen H., Fang B., Hu Z. (2007). Simultaneous
HPLC Determination of
Four Key Metabolites in the Metabolic Pathway for Production of 1,3-Propanediol
from Glycerol. Chromatographia.

[29] Liang D., Gao J., Sun H., Chen P., Hou Z., Zheng X. (2011). Selective
Oxidation of Glycerol with Oxygen in a Base-Free Aqueous Solution
over MWNTs Supported Pt Catalysts. Appl. Catal.
B Environ..

[30] Demirel-Gülen S., Lucas M., Claus P. (2005). Liquid Phase Oxidation
of Glycerol
over Carbon Supported Gold Catalysts. Catal.
Today.

[31] Demirel S., Lehnert K., Lucas M., Claus P. (2007). Use of Renewables
for
the Production of Chemicals: Glycerol Oxidation over Carbon Supported
Gold Catalysts. Appl. Catal. B Environ..

[32] Liebminger S., Siebenhofer M., Guebitz G. (2009). Oxidation of Glycerol
by 2,2,6,6-Tetramethylpiperidine-N-Oxyl
(TEMPO) in the Presence of Laccase. Bioresour.
Technol..

[33] Ketchie W. C., Fang Y. L., Wong M. S., Murayama M., Davis R. J. (2007). Influence
of Gold Particle Size on the Aqueous-Phase Oxidation of Carbon Monoxide
and Glycerol. J. Catal..

[34] Zope B. N., Davis R. J. (2011). Inhibition of Gold
and Platinum Catalysts by Reactive
Intermediates Produced in the Selective Oxidation of Alcohols in Liquid
Water. Green Chem..

[35] Kwon Y., Koper M. T. M. (2010). Combining Voltammetry
with HPLC: Application to Electro-Oxidation
of Glycerol. Anal. Chem..

[36] Augugliaro V., El Nazer H. A. H., Loddo V., Mele A., Palmisano G., Palmisano L., Yurdakal S. (2010). Partial Photocatalytic Oxidation
of Glycerol in TiO2 Water Suspensions. Catal.
Today.

[37] Carrettin S., McMorn P., Johnston P., Griffin K., Kiely C. J., Attard G. A., Hutchings G. J. (2004). Oxidation
of Glycerol Using Supported
Gold Catalysts. Top. Catal..

[38] Hu W., Knight D., Lowry B., Varma A. (2010). Selective Oxidation
of Glycerol to Dihydroxyacetone over Pt–Bi/C Catalyst: Optimization
of Catalyst and Reaction Conditions. Ind. Eng.
Chem. Res..

[39] Santana C. S., Gjonaj E., Garcia A. C. (2024). Effect of Iron Impurities on the
Electrochemical Oxidation of Glycerol on Ni­(OH)­2/NiOOH Electrodes. ChemElectroChem..

[40] Zhou Y., Shen Y., Piao J. (2018). Sustainable Conversion of Glycerol
into Value-Added Chemicals by Selective Electro-Oxidation on Pt-Based
Catalysts. ChemElectroChem..

[41] Zhou Y., Shen Y., Xi J., Luo X. (2019). Selective Electro-Oxidation
of Glycerol to Dihydroxyacetone by PtAg Skeletons. ACS Appl. Mater. Interfaces.

[42] Zhou Y., Shen Y., Luo X. (2020). Optimizing
the Activity and Selectivity
of Glycerol Oxidation over Core-Shell Electrocatalysts. J. Catal..

[43] Garcia A. C., Kolb M. J., van Nierop y Sanchez C., Vos J., Birdja Y. Y., Kwon Y., Tremiliosi-Filho G., Koper M. T. M. (2016). Strong Impact of Platinum Surface Structure on Primary
and Secondary Alcohol Oxidation during Electro-Oxidation of Glycerol. ACS Catal..

[44] Trafela Š., Zavašnik J., Šturm S., Rožman K. Ž. (2019). Formation of a Ni­(OH)­2/NiOOH Active
Redox Couple on Nickel Nanowires for Formaldehyde Detection in Alkaline
Media. Electrochim. Acta.

[45] Vedharathinam V., Botte G. G. (2014). Experimental Investigation
of Potential Oscillations
during the Electrocatalytic Oxidation of Urea on Ni Catalyst in Alkaline
Medium. J. Phys. Chem. C.

[46] Habibi B., Delnavaz N. (2016). Electrooxidation of
Glycerol on Nickel and Nickel Alloy
(Ni-Cu and Ni-Co) Nanoparticles in Alkaline Media. RSC Adv..

[47] Luo H., Yukuhiro V. Y., Fernández P. S., Feng J., Thompson P., Rao R. R., Cai R., Favero S., Haigh S. J., Durrant J. R., Stephens I. E. L., Titirici M. M. (2022). Role of Ni in PtNi
Bimetallic Electrocatalysts for Hydrogen and Value-Added Chemicals
Coproduction via Glycerol Electrooxidation. ACS Catal..

[48] ICH Topic Q 2 (R1) Validation of Analytical Procedures: Text and Methodology. Step 5 NOTE FOR GUIDANCE ON VALIDATION OF ANALYTICAL PROCEDURES: TEXT AND METHODOLOGY (CPMP/ICH/381/95); European Medicines Agency, November 1994.

[49] Ratner B. (2009). The Correlation
Coefficient: Its Values Range between 1/1, or Do They. J. Targeting, Meas. Anal. Mark..

[50] Wu J., Yang X., Gong M. (2022). Recent Advances
in Glycerol Valorization
via Electrooxidation: Catalyst, Mechanism and Device. Chin. J. Catal..

[51] Vehrenberg J., Baessler J., Decker A., Keller R., Wessling M. (2023). Paired Electrochemical
Synthesis of Formate via Oxidation of Glycerol and Reduction of CO2
in a Flow Cell Reactor. Electrochem. commun..

[52] Chen W., Zhang L., Xu L., He Y., Pang H., Wang S., Zou Y. (2024). Pulse Potential Mediated Selectivity
for the Electrocatalytic Oxidation of Glycerol to Glyceric Acid. Nat. Commun..

[53] Jiang J., Kucernak A. (2002). Nanostructured Platinum as an Electrocatalyst
for the
Electrooxidation of Formic Acid. J. Electroanal.
Chem..

[54] Labata M. F., Li G., Ocon J., Chuang P. Y. A. (2021). Insights
on Platinum-Carbon Catalyst
Degradation Mechanism for Oxygen Reduction Reaction in Acidic and
Alkaline Media. J. Power Sources.

[55] McCrory C. C. L., Jung S., Ferrer I. M., Chatman S. M., Peters J. C., Jaramillo T. F. (2015). Benchmarking
Hydrogen Evolving Reaction and Oxygen
Evolving Reaction Electrocatalysts for Solar Water Splitting Devices. J. Am. Chem. Soc..

[56] Morin M. C., Lamy C., Léger J. M., Vasquez J. L., Aldaz A. (1990). Structural
Effects in Electrocatalysis. Oxidation of Ethanol on Platinum Single
Crystal Electrodes. Effect of PH. J. Electroanal.
Chem..

[57] Sun S. G., Clavilier J. (1987). Electrochemical Study on the Poisoning
Intermediate
Formed from Methanol Dissociation at Low Index and Stepped Platinum
Surfaces. J. Electroanal. Chem..

[58] Goetz M. K., Usman E., Choi K. S. (2023). Understanding and Suppressing C-C
Cleavage during Glycerol Oxidation for C3 Chemical Production. ACS Catal..

[59] Shubair A., Houache M. S. E., Mousavi
M S. S., Botton G. A., Baranova E. A. (2022). Electrolysis
of Glycerol to Value-Added Chemicals in Alkaline Media. J. Chem. Technol. Biotechnol..

[60] Balog Á., Kecsenovity E., Samu G. F., He J., Fekete D., Janáky C. (2024). Paired Photoelectrochemical
Conversion of CO2/H2O and
Glycerol at High Rate. Nat. Catal..

[61] Kimura H. (1993). Selective
Oxidation of Glycerol on a Platinum-Bismuth Catalyst by Using a Fixed
Bed Reactor. Appl. Catal. A Gen..

[62] Kwon Y., Birdja Y., Spanos I., Rodriguez P., Koper M. T. M. (2012). Highly Selective Electro-Oxidation of Glycerol to Dihydroxyacetone
on Platinum in the Presence of Bismuth. ACS
Catal..

[63] Naish P. J., Hartwell S. (1988). Exponentially Modified Gaussian Functions—A
Good Model for Chromatographic Peaks in Isocratic HPLC?. Chromatographia.

[64] Stevenson P. G., Guiochon G. (2013). Cumulative Area of
Peaks in a Multidimensional High
Performance Liquid Chromatogram. J. Chromatogr.
A.

[65] De
Lima R. B., Paganin V., Iwasita T., Vielstich W. (2003). On the Electrocatalysis
of Ethylene Glycol Oxidation. Electrochim. Acta.

[66] Tonelli D., Ballarin B., Berrettoni M., Trevisani M. (2003). Electrochemical
Study of Mannitol Oxidation at Nickel Oxide Electrode. Collect. Czechoslov. Chem. Commun..

[67] Creus J., Miola M., Pescarmona P. P. (2023). Unravelling
and Overcoming the Challenges
in the Electrocatalytic Reduction of Fructose to Sorbitol. Green Chem..

[68] International Organization for Standardization . Borosilicate glass 3.3 - Properties - ISO 3585:1991. https://cdn.standards.iteh.ai/samples/8994/8c84cf66312e4da1a1121be40e5607b2/ISO-3585-1991.pdf (accessed 2024–11–20).

[69] International Organization for Standardization . Glass - Resistance to attack by boiling aqueous solution of mixed alkali - Method of test and classification - ISO 695:1991. https://cdn.standards.iteh.ai/samples/4895/7aa726421ce94544aa1394ce345bb271/ISO-695-1991.pdf (accessed 2024–11–20).

