# Supplementary material for: Simple and Effective HPLC Method for Elucidating Glycerol Oxidation Products
Source: ACS Meas Sci Au. 2025 May 31;5(3):367–77. doi: 10.1021/acsmeasuresciau.5c00024 (PMC12183600; doi:10.1021/acsmeasuresciau.5c00024)
Supplement: Supplementary file 1 [file tg5c00024_si_001.pdf]

Supporting Information

Simple and Effective HPLC Method for Elucidating Glycerol Oxidation Products

Eva Ng [a], Camilo A. Mesa[*a,b], Elena Mas-Marzá,[a] Sixto Giménez[*a].

[a] Institute of Advanced Materials, Universitat Jaume I, 12006, Castelló, Spain.

[b] Catalan Institute of Nanoscience and Nanotechnology (ICN2), CSIC, Barcelona Institute of Science and Technology, UAB Campus, 08193 Bellaterra, Barcelona, Spain

Corresponding author: Camilo A. Mesa (cmesa@uji.es), Sixto Giménez (sjulia@uji.es)

1. *Faradaic efficiency and selectivity calculation*

Faradaic efficiency for each glycerol product is given by **Eq. S1**, where n_{HPLC} are the moles calculated using HPLC, n_{CA} are the theoretical moles calculated from the charge passed through the system and represented by **Eq. S2**. z is the number of electrons required to form a certain product (from **Eq. S3** to **Eq S8**)

$$FE = \frac{n_{HPLC}}{n_{CA}} \times 100 \qquad \textbf{Eq. S1}$$

$$n_{CA} = \frac{Q\ (C)}{\mathrm{z} \times 96485\ {}^{C}/_{mol\ e^-}} \qquad \textbf{Eq. S2}$$

Semi-reactions involved for Faradaic efficiencies calculation:

$$C_3H_8O_3 +\ 2\ OH^- \rightarrow\ C_3H_6O_3(GlyAld\ or\ DHA) +\ 2\ H_2O + 2\ e^- \qquad \textbf{Eq. S3}$$

$$C_3H_8O_3 +\ 5\ OH^- \rightarrow\ C_3H_5O_4{}^-(glycerate) +\ 4\ H_2O + 4\ e^- \qquad \textbf{Eq. S4}$$

$$C_3H_8O_3 + {}^{13}/_2\ OH^- \rightarrow\ {}^{3}/_2\ C_2H_3O_3{}^-(glycolate) +\ 5\ H_2O + 5\ e^- \qquad \textbf{Eq. S5}$$

$$C_3H_8O_3 +\ 11\ OH^- \rightarrow\ 3\ C_1H_1O_2{}^-(formate) +\ 8\ H_2O + 8\ e^- \qquad \textbf{Eq. S6}$$

$$C_3H_8O_3 + 14\ OH^- \rightarrow {}^3/_2\ C_2O_4^{2-}(\text{oxalate}) + 11\ H_2O + 11\ e^- \qquad \textbf{Eq. S7}$$

$$C_3H_8O_3 + 10\ OH^- \rightarrow C_3H_2O_5^{2-}(\text{tartronate}) + 8\ H_2O + 8\ e^- \qquad \textbf{Eq. S8}$$

Where, $GlyAld = glyceraldehyde$ and $DHA = dihydroxyacetone$

On the other hand, selectivity is expressed by **Eq. S9**, where n_{HPLC} are the moles from the HPLC quantification and n_i is the sum of all of the moles of products formed during GEOR (measured by HPLC).

$$Selectivity = \frac{n_{HPLC}}{\sum(n_i)} \times 100 \qquad \textbf{Eq. S9}$$

2. *Selection of HPLC conditions*

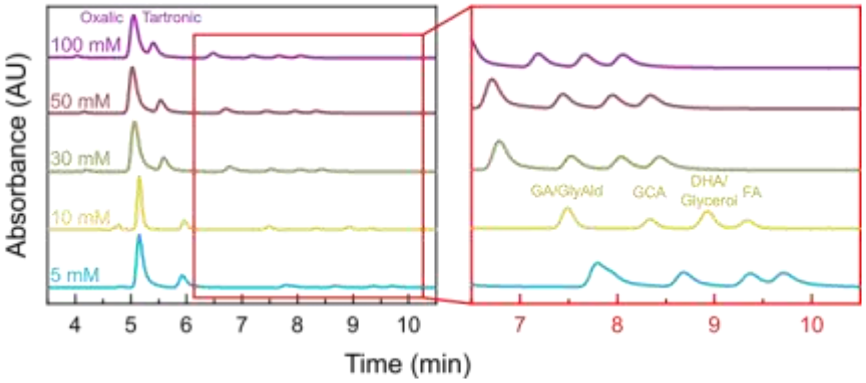

Figure S1. Chromatograms showing the mobile phase (H_2SO_4) concentration (5 -100 mM) effect towards the separation and resolution of solution containing oxalic, tartronic, glyceric, glycolic and formic acid along with glyceraldehyde and DHA, each at a concentration of 1.25 mM. Each product is shown at one condition as visual guide. It is worth noticing that H_2SO_4 concentration effect did not alter product's elution order. The operation conditions were 0.6 mL·min^{-1}, 50 ºC and an injection volume of 20 µL. Red rectangles represent a zoomed area.

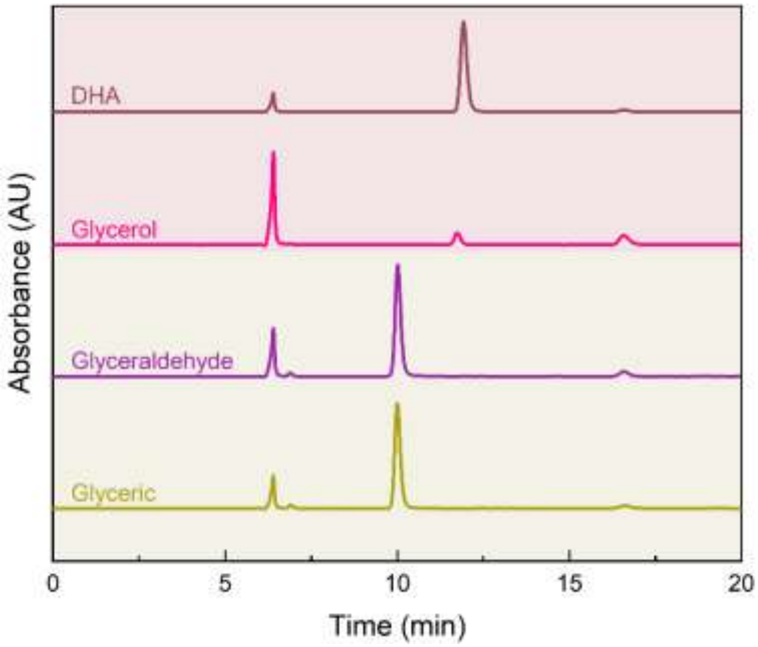

Figure S2. Chromatogram presenting overlapping peaks between glyceraldehyde and glyceric acid (green rectangle) at ~10 min and glycerol with DHA (pink rectangle) at ~11.5 min, using H_2SO_4 10 mM as a mobile phase.

Figure S3, shows the effect when acetonitrile is used as a mobile phase modifier. The purpose of this figure is to simply illustrate that acetonitrile can play an important role associated to the retention times. When the acetonitrile content increases, the chromatogram varies in a non-systematic way depending on the chemical nature of the product. For this reason and simplicity, each peak is not directly identified. Moreover, mobile phase composition along with other parameters such as temperature and flow rate represent are yet needed to find the optimal HPLC separation.

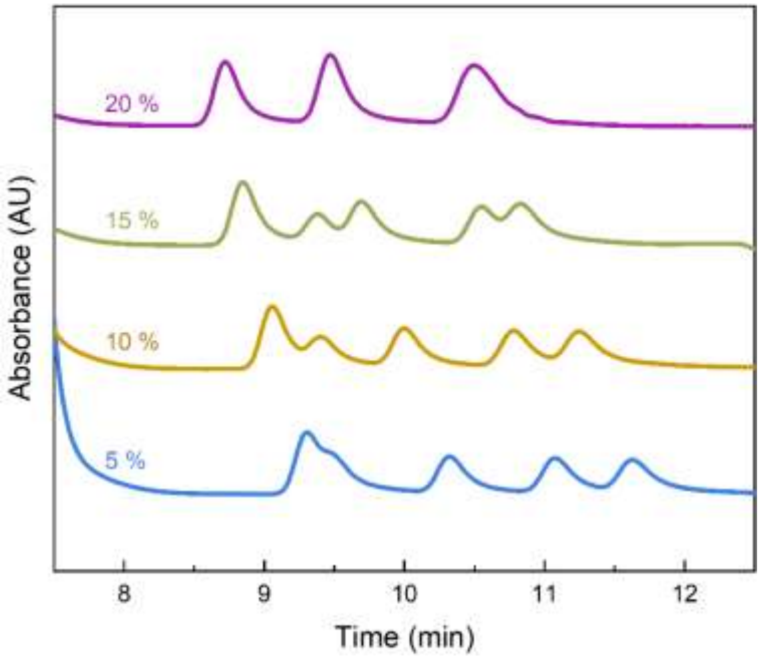

Figure S3. Effect of acetonitrile as a mobile phase modifier on the separation of a solution containing formic acid, DHA, glyceric acid, glyceraldehyde, glycerol and glycolic acid, each at a concentration of 1.25 mM and using 10 mM H_2SO_4 as the mobile phase.

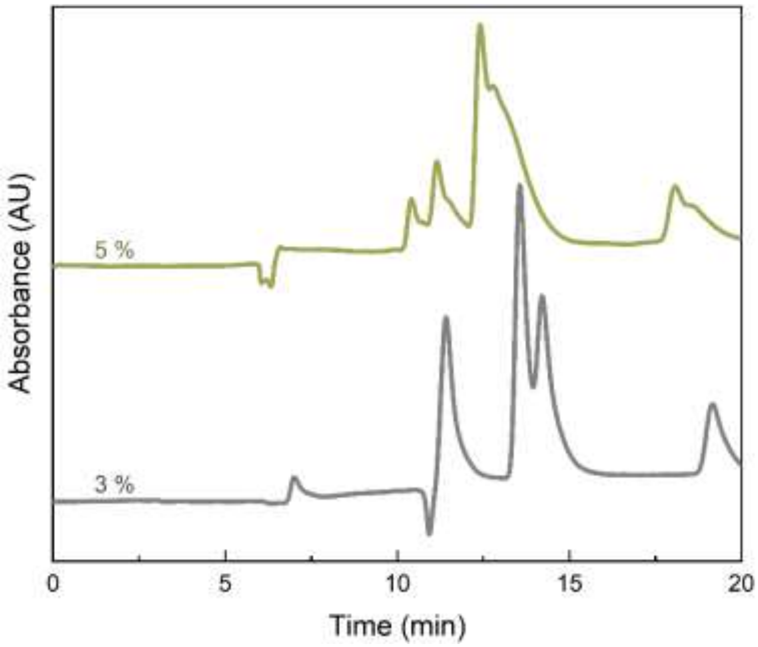

Figure S4. Effect of methanol as a mobile phase modifier on the separation of a solution containing formic acid, DHA, glyceric acid, glyceraldehyde, and glycolic acid, each at a concentration of 1.25 mM and using 10 mM H_2SO_4 as the mobile phase.

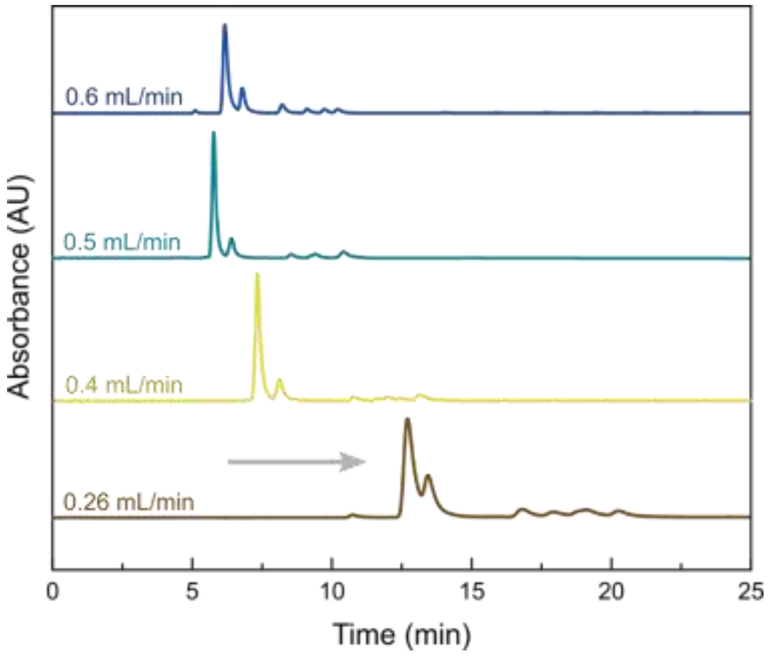

Figure S5. Flow rate effect in the separation and resolution of a solution containing oxalic acid, tartronic acid, formic acid, DHA, glyceric acid, glyceraldehyde, and glycolic acid, each at a concentration of 1.25 mM and using 10 mM H_2SO_4 as the mobile phase.

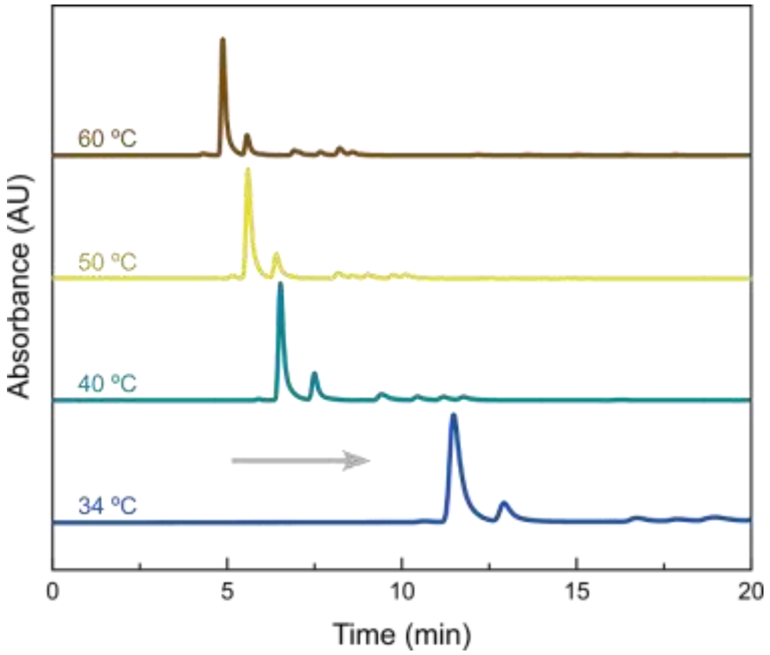

Figure S6. Temperature effect in the separation and resolution of a solution containing oxalic acid, tartronic acid, formic acid, DHA, glyceric acid, glyceraldehyde, and glycolic acid, each at a concentration of 1.25 mM and using 10 mM H_2SO_4 as the mobile phase. Volume of injection was 20 µL.

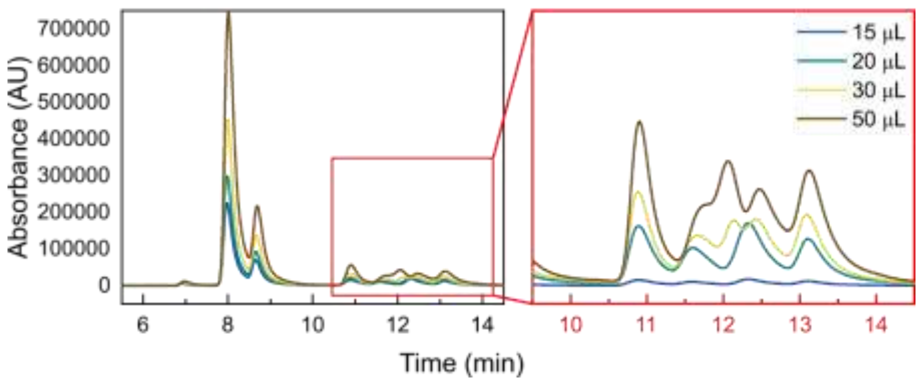

Figure S7. Volume of injection effect in the separation and resolution of a solution containing oxalic acid, tartronic acid, formic acid, DHA, glyceric acid, glyceraldehyde, and glycolic acid, each at a concentration of 1.25 mM and using 10 mM H_2SO_4 as the mobile phase at 0.6 mL min^{-1}. Red rectangles represent the zoomed area.

3. *Method validation*

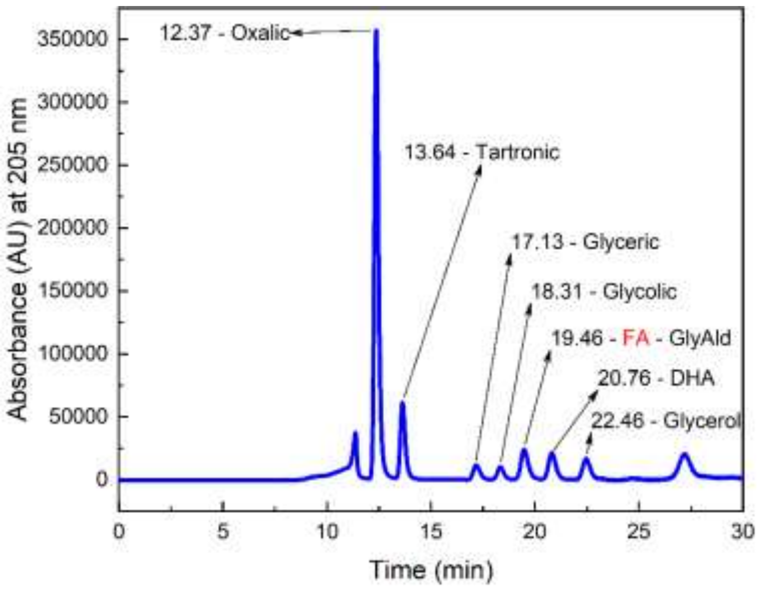

Figure S8. Chromatogram obtained from the analysis of analytical standards containing glycerol, oxalic acid, tartronic acid, glyceric acid, glycolic acid, formic acid, glyceraldehyde and DHA, each at a concentration of 5 mM. These chromatograms were collected using a flow rate of 0.26 mL min^{-1}, 20 µL volume of injection and 34 ºC for the column temperature. Mobile phase was 70:30 H_2SO_4

(10 mM) and acetonitrile. Signals at ~11 min and ~27 min are salt- and water-related peaks, respectively.

Table S1. Determined slopes for GlyAld standard at different wavelengths.

**Glyceraldehyde**	
Wavelength (nm)	*(Slope ± error)*
195	20349 ± 36
205	12566 ± 50
220	2303 ± 21
270	368 ± 7
300	506 ± 4

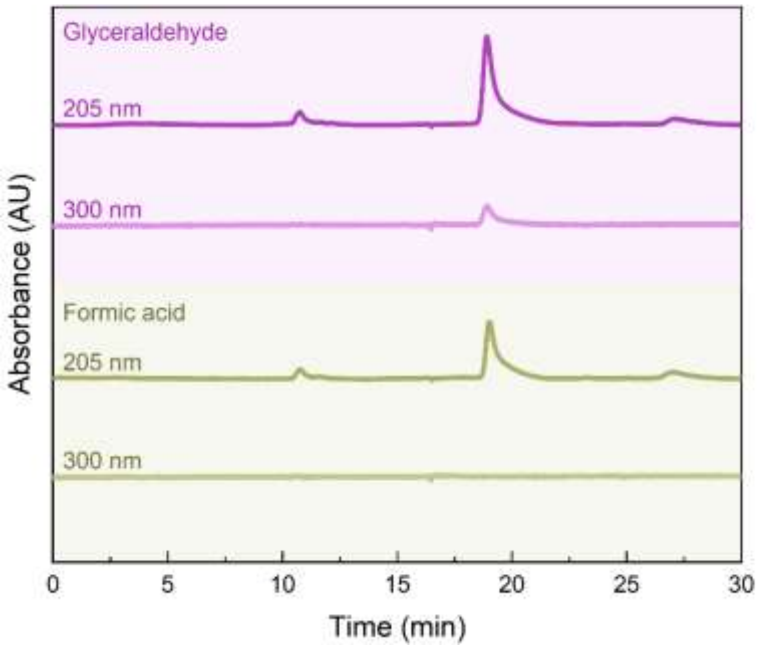

Figure S9. Chromatograms for glyceraldehyde and formic acid at 205 nm and 300 nm, both with a concentration of 5 mM each. The operational parameters were a flow rate of 0.26 mL min^{-1}, 20 µL volume of injection and 34 ºC for the column temperature. Mobile phase was 30 % acetonitrile and 70 % H_2SO_4 10 mM.

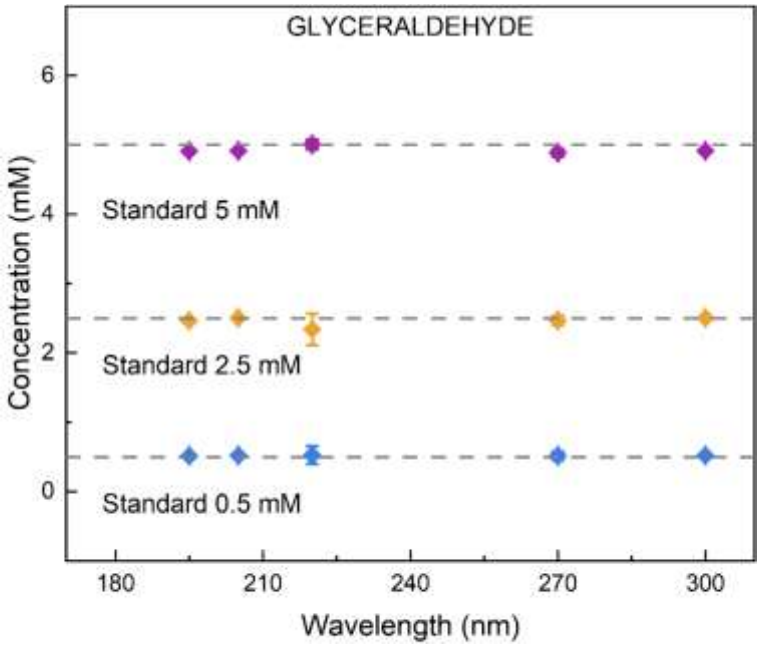

Figure S10. Glyceraldehyde standard concentrations as a function of different wavelengths. Each data point includes error bars representing the variability at each level.

Table S2. Absolute error values referred in **Figure 3**.

**Concentration**		
Experimental (E)	*Theoretical (T)*	*Absolute error (%)*
0.11	0.1	7
0.51	0.5	3
0.99	1.0	1
2.53	2.5	1
5.04	5.0	0.7

4. *Application of the method: Electrochemical measurements and Faradaic Efficiencies*

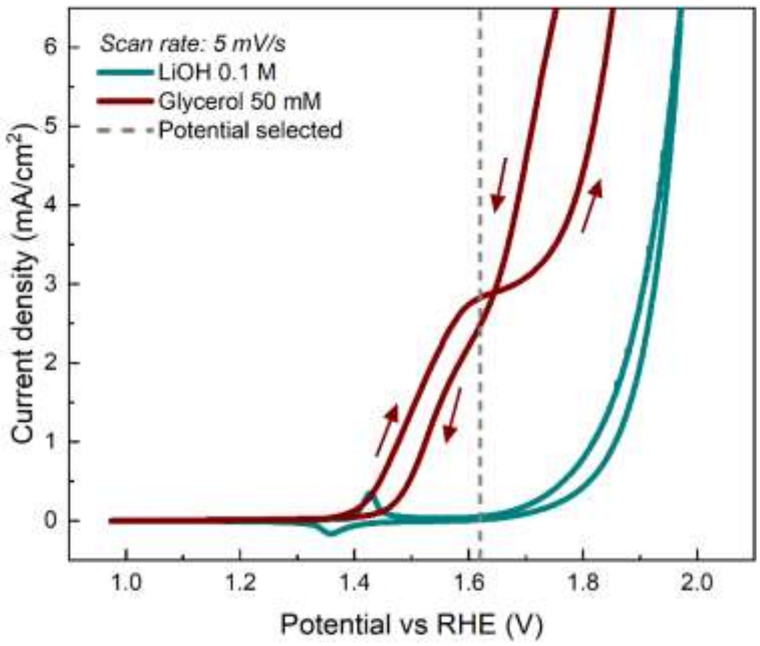

Figure S11. Cyclic voltammetry of a Ni-based electrode in 0.1 M LiOH (green) and 50 mM glycerol (red), measured at 5 mV s^{-1}.

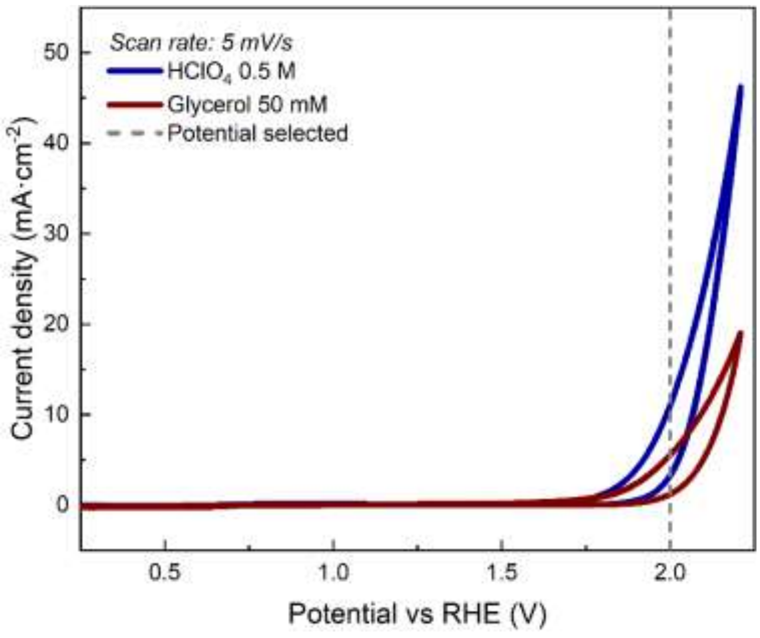

Figure S12. Cyclic voltammetry of a Pt-based electrode in 0.5 M $HClO_4$ (blue) and 50 mM glycerol (red), measured at 5 mV s^{-1}.

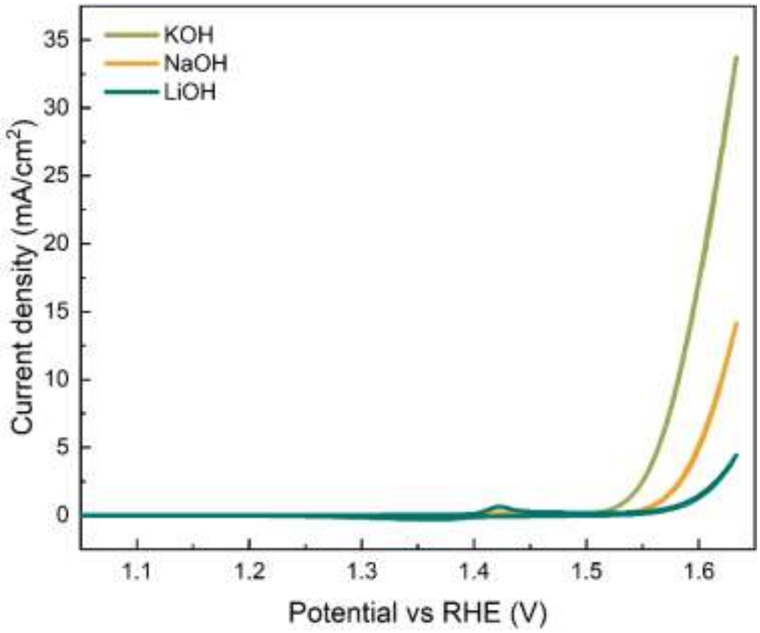

Figure S13. Cation effect towards Oxygen Evolution Reaction (OER) measured by Cyclic Voltammetry, using a scan rate of 5 mV s^{-1}. Data reproduced from Figure S4 in the Supporting Information of Ng et al. 2024.[1]

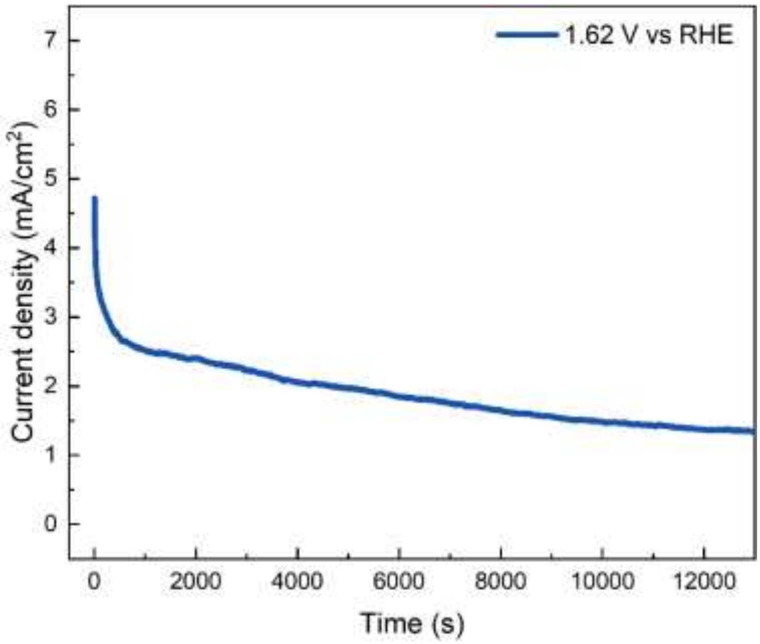

Figure S14. Chronoamperometric curve at 1.62 V vs RHE until a total of 50 C was passed in a solution containing 0.1 M LiOH + 50 mM glycerol using Ni-based electrode.

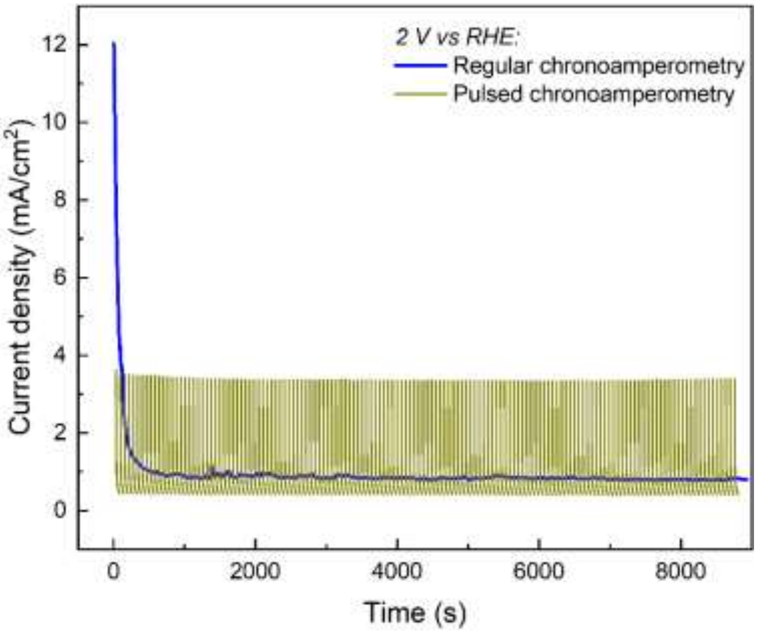

Figure S15. Comparison between a pulsed chronoamperometry (green curve) at 2 V vs RHE for 50 s and 10 s of rest at OCP and a regular chronoamperometry (blue curve), during 2 hours in a solution containing 0.5 M $HClO_4$ + 50 mM glycerol using Pt-based electrode.

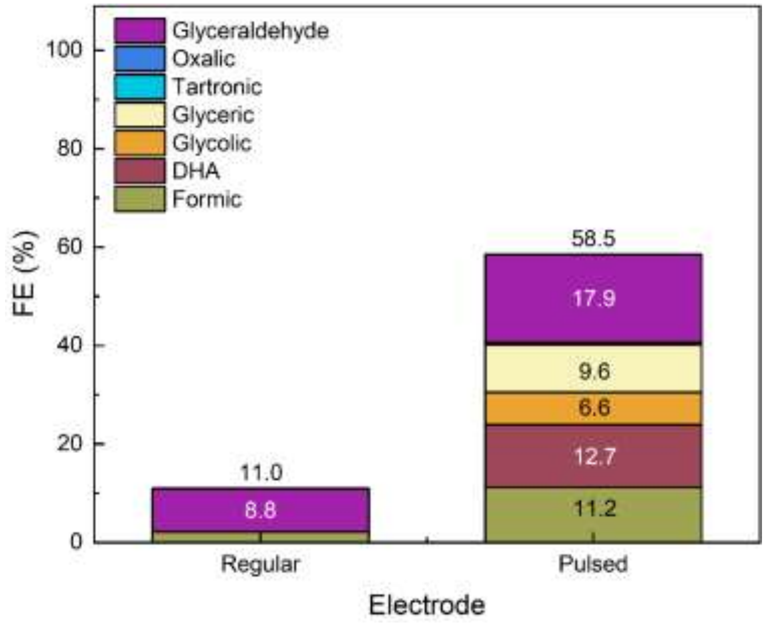

Figure S16. Product distribution represented in terms of Faradaic Efficiency (FE) after applying 2 V vs RHE using Pt foil as working electrode comparing regular and pulsed chronoamperometry. The percentages above the bars represent the total (FE) while inside the bars, they refer to the different products.

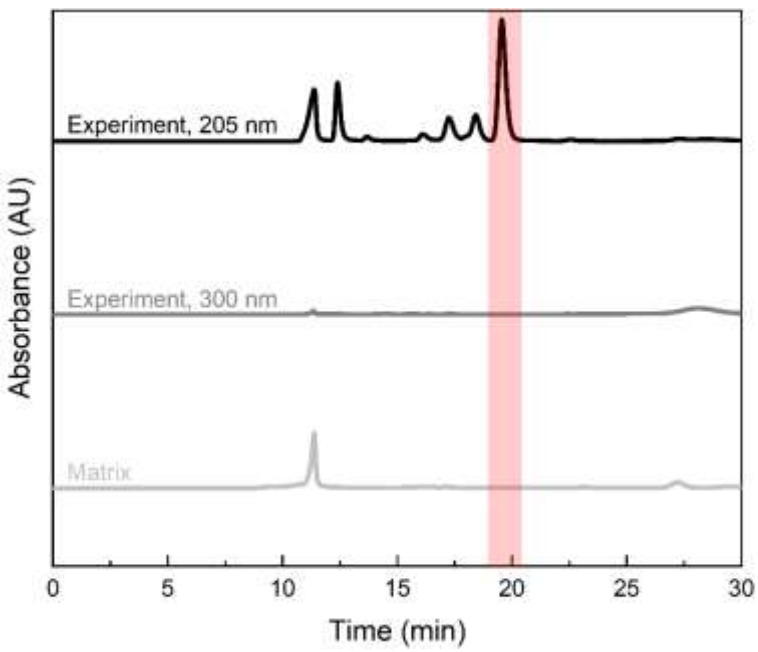

Figure S17. Collected chromatogram after the GEOR using Ni-foil as catalyst at 1.62 V vs RHE. At 205 nm all the products obtained are shown and at 300 nm glyceraldehyde detection and quantification is performed. Matrix (LiOH 0.1 + glycerol 50 mM, acidified with H_2SO_4 0.5 M) chromatogram measured at 205 nm is included as reference. The red box highlights glyceraldehyde retention time. The operational parameters were a flow rate of 0.26 mL min^{-1}, 20 µL volume of injection and 34 ºC for the column temperature. Mobile phase was 30 % acetonitrile and 70 % H_2SO_4 10 mM.

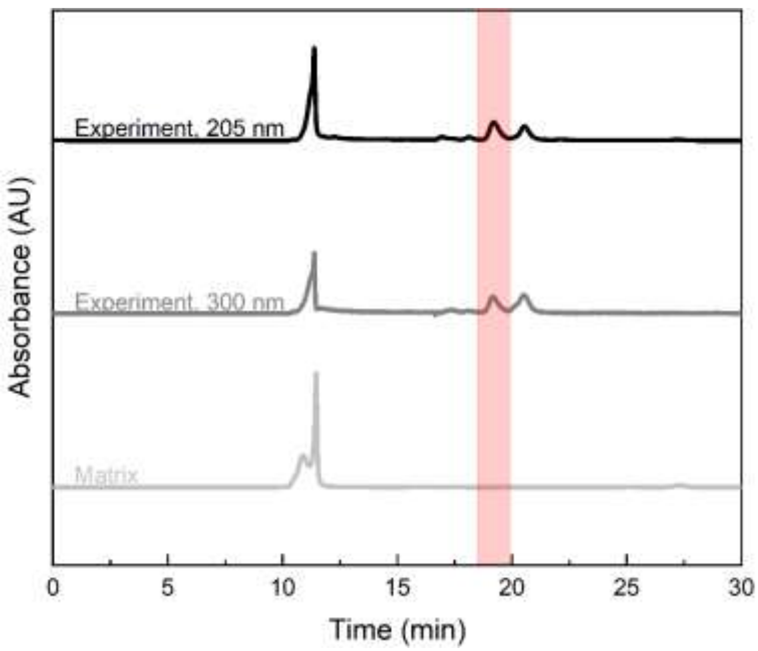

Figure S18. Collected chromatogram after the GEOR using Pt-foil as catalyst at 2 V vs RHE. At 205 nm all the products obtained are shown and at 300 nm glyceraldehyde detection and quantification is performed. Matrix ($HClO_4$ 0.5 + glycerol 50 mM, acidified with H_2SO_4 0.5 M) chromatogram measured at 205 nm is included as reference. The red box highlight glyceraldehyde retention time. The operational parameters were a flow rate of 0.26 mL min^{-1}, 20 µL volume of injection and 34 ºC for the column temperature. Mobile phase was 30 % acetonitrile and 70 % H_2SO_4 10 mM.

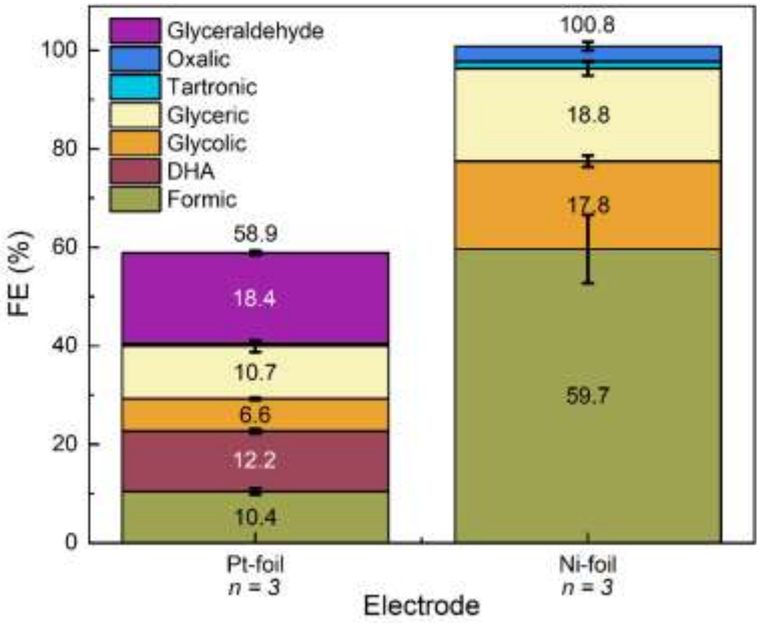

Figure S19. Product distribution represented in terms of Faradaic Efficiency (FE). The percentages above the bars represent the total (FE) while inside the bars, they refer to the different products. n refers to the repetitions of the experiment.

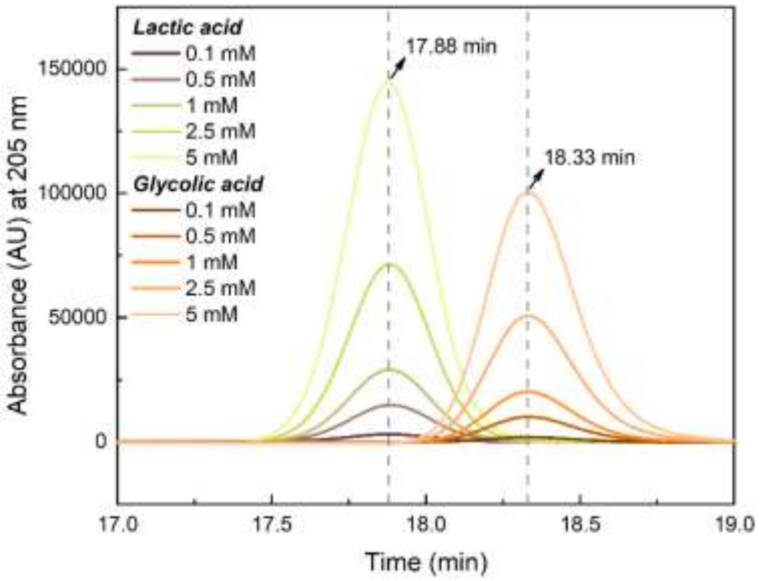

Figure S20. Calibration chromatograms of lactic acid and glycolic acid at various concentrations (0.1-5 mM) showing their relative retention times under the optimized HPLC conditions. These chromatograms were collected using a flow rate of 0.26 mL min^{-1}, 20 µL volume of injection and 34 ºC for the column temperature. Mobile phase was 70:30 H_2SO_4 (10 mM) and acetonitrile.

References

(1) Ng, E.; Mesa, C. A.; Más-Marzá, E.; Giménez, S. Current-Dependent Product Distribution and Reaction Mechanisms of Glycerol Electrooxidation on Nickel. *ChemElectroChem* **2024**, *202400534*, 1–10. https://doi.org/10.1002/celc.202400534.
